# Identification of Potential COX-2 Inhibitors for the Treatment of Inflammatory Diseases Using Molecular Modeling Approaches

**DOI:** 10.3390/molecules25184183

**Published:** 2020-09-12

**Authors:** Pedro H. F. Araújo, Ryan S. Ramos, Jorddy N. da Cruz, Sebastião G. Silva, Elenilze F. B. Ferreira, Lúcio R. de Lima, Williams J. C. Macêdo, José M. Espejo-Román, Joaquín M. Campos, Cleydson B. R. Santos

**Affiliations:** 1 Graduate Program in Innovation Pharmaceutical, Federal University of Amapá, 68903-419 Amapá-AP, Brazil; pedro.henrique.fauro@gmail.com (P.H.F.A.); elenilze@yahoo.com.br (E.F.B.F.); williamsmacedo@yahoo.com.br (W.J.C.M.); 2 Laboratory of Modeling and Computational Chemistry, Department of Biological and Health Sciences, Federal University of Amapá, 68902-280 Macapá-AP, Brazil; ryanquimico@hotmail.com (R.S.R.); jorddynevescruz@gmail.com (J.N.d.C.); luciorolima@gmail.com (L.R.d.L.); 3 Campus Abaetetuba, Universidade Federal do Para, Ramal Manoel de Abreu, s/n-Mutirão, Abaetetuba, 68440-000 Pará, Brazil; profsebastiaogs@gmail.com; 4 Laboratory of Organic Chemistry and Biochemistry, University of State of Amapá, 68900-070 Macapá-AP, Brazil; 5 Laboratory of Molecular Modeling and Simulation System, Federal Rural University of Amazônia, Rua João Pessoa, 121, Capanema, 68700-030 Pará-PA, Brazil; 6 Department of Pharmaceutical Organic Chemistry, Faculty of Pharmacy, Biosanitary Institute of Granada (Ibs.GRANADA), Campus of Cartuja s/n, University of Granada, 18071 Granada, Spain; josemanuelespejo@correo.ugr.es (J.M.E.-R.); jmcampos@ugr.es (J.M.C.)

**Keywords:** in silico, COX-2 inhibitors, molecular modeling

## Abstract

Non-steroidal anti-inflammatory drugs are inhibitors of cyclooxygenase-2 (COX-2) that were developed in order to avoid the side effects of non-selective inhibitors of COX-1. Thus, the present study aims to identify new selective chemical entities for the COX-2 enzyme via molecular modeling approaches. The best pharmacophore model was used to identify compounds within the ZINC database. The molecular properties were determined and selected with Pearson’s correlation for the construction of quantitative structure–activity relationship (QSAR) models to predict the biological activities of the compounds obtained with virtual screening. The pharmacokinetic/toxicological profiles of the compounds were determined, as well as the binding modes through molecular docking compared to commercial compounds (rofecoxib and celecoxib). The QSAR analysis showed a fit with R = 0.9617, R^2^ = 0.9250, standard error of estimate (SEE) = 0.2238, and F = 46.2739, with the tetra-parametric regression model. After the analysis, only three promising inhibitors were selected, **Z-964**, **Z-627**, and **Z-814**, with their predicted pIC_50_ (−log IC_50_) values, **Z-814** = 7.9484, **Z-627** = 9.3458, and **Z-964** = 9.5272. All candidates inhibitors complied with Lipinski’s rule of five, which predicts a good oral availability and can be used in in vitro and in vivo tests in the zebrafish model in order to confirm the obtained in silico data.

## 1. Introduction

Inflammatory processes stand out for their pathophysiological aspect, as they are caused by pathogenic microorganisms, such as viruses, bacteria, fungi, and parasites that invade host cells to reproduce, resulting in a complex and heterogeneous group of diseases that cause morbidity and mortality to those affected by them. At the same time, the need for specific attention to protect the integrity of human organisms from harmful or exogenous agents is emphasized [1].

Inflammatory mediation is regulated by the action of neutrophils, mast cells, eosinophils, macrophages, dendritic cells, and epithelial cells. It is a complex process involving vasodilation, chemotaxis, and increased permeability. Sensors called Toll-like receptors (TLR) recognize the products of pathogens, such as endotoxins and bacterial DNA that are located in the plasma membrane and in the endosomes, and they are capable of detecting intra and extracellular microorganisms [2].

When an injury occurs, platelets release complement proteins, where mast cells degranulate releasing histamine (vasodilation) and serotonin (cell permeability and diapedesis), where neutrophils are activated and migrate to the site of action induced by chemokines. Neutrophils phagocytose pathogenic organisms releasing mediators and attracting macrophages, increasing the release of pro-inflammatory mediators (prostaglandins and leukotrienes) and cytokines (interleukin 1 (IL-1), interleukin 6 (IL-6) and tumor necrosis factor (TNFα)). When cells are activated, the arachidonic acid (AA) of the cell membrane is converted by enzymes for the synthesis of prostaglandins and leukotrienes.

**Figure 1:**
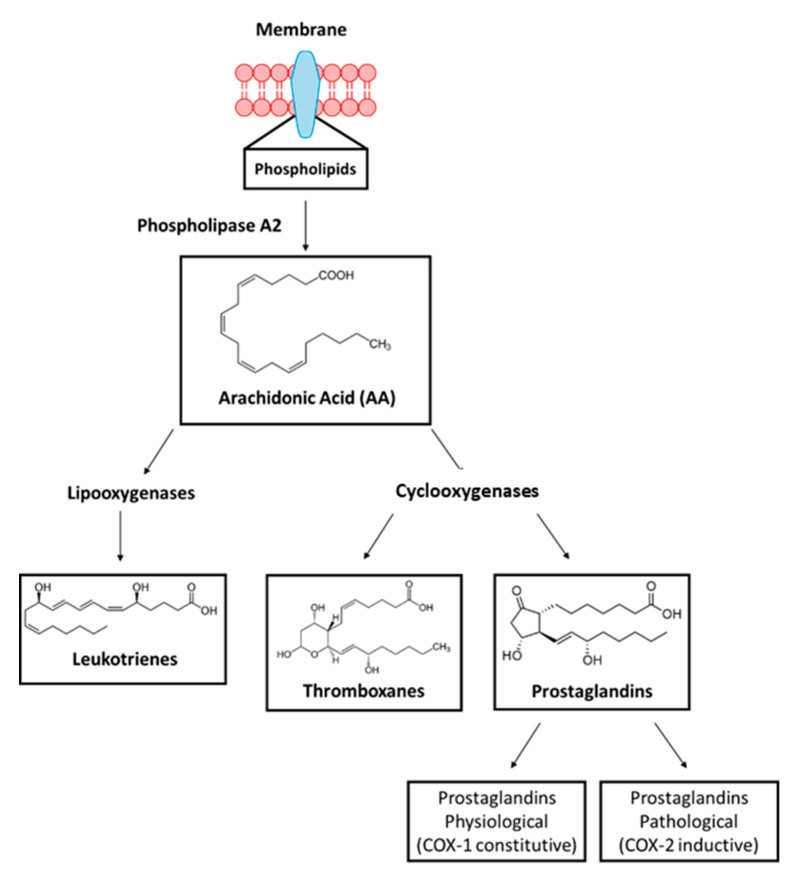
Cascade of arachidonic acid.

In one of the stages of the inflammatory process, prostaglandins, also called eicosanoids, are synthesized by triggering various stimuli that activate cell membrane receptors, when coupled with a regulatory protein that results in the activation of phospholipase A2 or with an increase in the concentration of Ca^+2^. This type of enzyme hydrolyzes membrane phospholipids, consequently releasing the cascade of arachidonic acid, which is a substrate for the synthesis of physiopatological and inductive prostaglandins; see Figure 1 below [3].

Prostaglandins-endoperoxide synthase (PTGS) are known as cyclooxygenases responsible for the synthesis of prostaglandins that are percussive biologically active molecules. Figure 1 shows that the conversion of AA into signaling molecules takes place in 2 moments. (i) The enzyme cyclooxygenase-1 (COX-1, PTGS1) catalyzes the addition of two free oxygen atoms to form the 1,2-dioxane bridge and a functional group to form prostaglandin G2 (endoperoxide (PGG2)). (ii) COX-2 (PTGS2) reduces the peroxide functional group to a secondary alcohol, forming prostaglandin H2 (PGH2). PGG2 and PGH2 are unstable, but they are precursors for the formation of other prostaglandins (PGE2, PGD2, PGF2), prostacyclin (PGl2), and thromboxane A2 (TXA2), which are commonly called eicosanoids.

Currently, it is known that two genes express two distinct, but similar, isoforms of COX. These enzymes catalyze the biosynthesis of prostaglandins and thromboxanes by reducing arachidonic acid; they are called COX-1 and COX-2, with similar general protein structures that essentially catalyze the same reaction. COX-1 is an enzyme considered to be constitutive; it is part of the homeostatic maintenance of various processes of the human organism, and it is present in most tissues, including stomach, kidney, and coronary arteries [4].

On the other hand, the COX-2 enzyme is considered inductive; that is, it expresses the inflammatory process in cells. The understanding of the COX inhibition process has allowed and still allows the development of new perspectives in relation to the therapeutic targets and the synthetic drugs produced, so that they are as selective and smooth as possible, corroborating their adverse effects [4,5]. Widely prescribed non-steroidal anti-inflammatory drugs (NSAIDs) are a class of drugs used to treat pain, fever, and inflammation. Since 1893, when acetylsalicylic acid was produced, NSAIDs have become the most accepted and prescribed drugs. However, it was not until 1971 that John Vane clarified the mechanism of action of these drugs: They inhibit cyclooxygenase (COX), preventing consequently the synthesis of prostaglandins [6].

Non-selective or traditional NSAIDs inhibit both isoforms (1 and 2), but COX-1 inhibition is the main cause of increased gastrointestinal bleeding or ulcer formation, abdominal pain, and dyspepsia, including indomethacin, naproxen, and ibuprofen. On the other hand, COX-2 inhibition, inducing pro-inflammatory processes, plays a role in pain relief with reduced gastrointestinal effects, which is usually expressed by the coxib class, among them rofecoxib, celecoxib, valdecoxib, and lumiracoxib [7].

The knowledge of the structures of COX-1 and COX-2 and their active sites constitute the fundamental basis for the development of more specific inhibitors for COX-2 and for the elaboration of studies of the structure–activity relationship of these products. During enzymatic activity, arachidonic acid binds to an Arg^120^ and to a Ser^530^. An electron transfers Tyr^385^ to an oxidized heme, which is also bound within the enzyme, initiating the reaction cyclooxygenase. Several studies have attempted to elucidate how and where non-steroidal anti-inflammatory drugs act on cyclooxygenase to block prostaglandin synthesis. Within the hydrophobic channel of COX, an amino acid difference at position 523 (isoleucine in COX-1 and valine in COX-2) can be of critical importance in the selectivity of several drugs [8].

One of the main drugs used, with a selective effect to inhibit the pro-inflammatory effect of COX-2, was rofecoxib, which would have the potential for treatment without side effects such as ulcers and gastrointestinal problems. Studies by Bombardier et al. 2000 [5] reported that when compared to a 500 mg dose of naproxen per day for the same period, the incidence of efficacy was equivalent, but the administration of rofecoxib had less side effects such as bleeding gastric and duodenal ulcers. However, it was observed that the toxicological effect of rofecoxib, with a daily 50 mg dose for 9 consecutive months, doubled the acute myocardial infarction and strokes [8].

At the current stage of knowledge, in which the binding sites for specific inhibitors in COX-2 have already been described, and the three-dimensional protein structure of the enzyme is clearly established, the use of modern molecular modeling techniques should be able to triate new compounds of high affinity and specificity, but probably without the presence of the sulfonamide and sulfone groups of the second-generation compounds seen previously, thus representing the birth of a third generation of specific COX-2 inhibitors [9].

The process from a biological target project to a new drug discovery can take an average of 10 years or more, and the computational chemistry comes with an excellent direction in the rational planning of drugs, with already countless cases of success involving or using computer simulations citing as an example the main factors: losartan, atorvastatin, and celecoxib.

Mathematical analyses accompany in silico studies in order to enable the reduction of costs and time to obtain positive results, observing the molecular structures and their possible affinities with the therapeutic target, using the quantitative structure–activity relationship (QSAR). This method aims to build parametric models for predicting inhibitory activity (IC_50_) correlated with dependent variables such as physical–chemical, biological, and toxicological properties [10].

In these analyses, reduction filters are applied to the models used to predict inhibitory activity, correlating the molecule structures with their activity and toxicological potential, and comparing them with a positive control. In 2016, Brick et al. [10] applied the QSAR analysis to identify new antimalarial inhibitors from 1H-Imidazol-2-IL-Pyrimidine-4,6-Diamines, with reducing filters to eliminate descriptors that did not show correlation or information relevant to the process of statistical and toxicological analysis, beginning the screening with 107 compounds from the ZINC database and ending with four more promising compounds.

**Figure 2:**
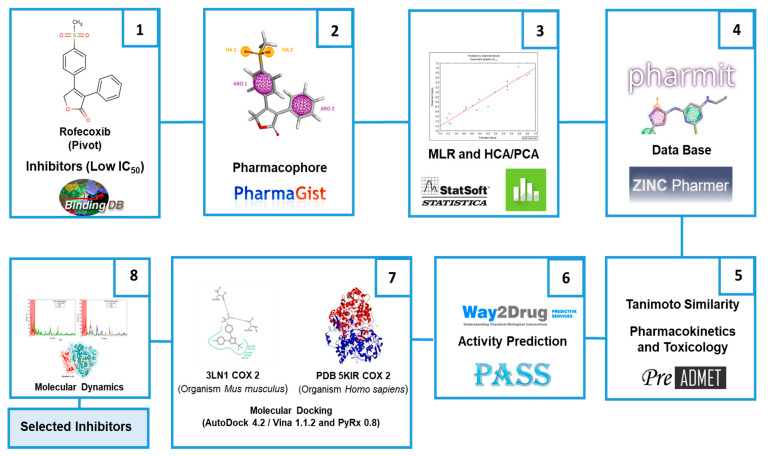
General scheme summarizing of the methodological steps.

In this context, one of the main types of studies in progress by the scientific community is the in silico and in vivo studies of inhibitors for the inflammatory processes to search for new selective molecules. In parallel, the QSAR study (quantitative structure–activity relationships) uses multiparametric models that interrelate biological activity with the physical–chemical properties of selected molecules in order to predict their inhibitory capacity against the inflammatory mechanism [10]. Therefore, the objective of this work is the virtual screening of analogs of rofecoxib (Vioox^®^), based on pharmacophore and QSAR analysis, understanding approaches and molecular modeling techniques through free software that is easily accessible by the scientific community, in parallel with the prediction of pharmacokinetic properties and toxicity that show the possible effectiveness of the selected structures, according to the methodological scheme presented in Figure 2 (see more details in the Materials and Methods section).

## 2. Results and Discussion

### 2.1. Molecular Optimization and QSAR Analysis

The structure of rofecoxib was selected as a pivot given its potential to mitigate the gastrointestinal effects compared to other selective drugs. Although this drug has the unwanted effect of acute myocardial infarction, the objective is to detect essential pharmacophore characteristics through virtual screening so that the selected promising structures have the same effectiveness. On the other hand, this is one of the only structures that presents the complexed structure in the Protein Data Bank (PDB) (5KIR, https://www.rcsb.org/) for the *Homo sapiens* organism, bringing the results of an ideality in front of the human organism.

The 32 molecules (Rofecoxib as a pivot) for the analysis were selected in the BindingDB database (https://www.bindingdb.org/bind/index.jsp) obeying an increasing order of IC_50_, with specific activity related to COX-2 and the *Homo sapiens* organism, in addition to not repeating inhibitory activity values, which could impact false-positive results through a straight line adjustment facilitated by of statistical analysis.

The molecular optimization values are shown in Table S1. The overlapping process was carried out by selecting molecules with the lowest energy value (PM3), since the optimization of molecular structures aims to bring the real structure closer to the energy minimum conformation, and with the observed experimental data, the optimization time quantification aims to elucidate the computational cost, as it is an expensive and time-consuming process [11]. 

Later, they were submitted to the PharmaGist software (https://bioinfo3d.cs.tau.ac.il/PharmaGist) for the extraction of physicochemical properties and construction of structure–activity relationship models (QSAR). The characteristics were analyzed with the aid of the Statistica^®^ software, where the most relevant ones were used to predict the inhibitory activity as a function of the pIC_50_ value to decrease statistical inconsistencies. This software is capable of predicting the relationship between the inhibitor structure and its inhibitory activity, with a Pearson correlation cut-off of 0.4, obtaining a training set with n = 20 structures (methodology adopted by Santos, Cruz and Santos) [12,13,14]. Table 1 shows the selected descriptors. The atoms (ATM) characteristic presented the best correlation among all descriptors, with a value of 0.7651, allowing inferring that the number of atoms significantly interferes in the pIC_50_ responses of the selected molecules. However, it must be noted that the selected regression model is tetra-parametric, so the prediction analysis must take into account the contribution of each descriptor in the process of prediction of the inhibitory activity value, as is the case of aromatic characteristics (ARO) with a p value of 0.7358 and acceptors (ACC) with a p value of 0.6399, which also contributes to the prediction of the inhibitory activity.

This result can also be accompanied by the analysis of hierarchical cluster analysis (HCA) (Figure S1) performed with the aid of the Minitab^®^ Trial software, allowing observation of the similarity between the physical–chemical characteristics and the inhibitory activity of the respective molecules, corroborating with the data obtained by Pearson’s correlation. The characteristics of ATM and ARO show greater proximity to the predicted pIC_50_. The descriptor ACC is inversely proportional to the predicted pIC_50_ value, which indicates that the presence of hydrophilic groups capable of establishing hydrogen-bonding interactions can increase the inhibitory potential of the selected structures.

The ATM characteristic may not be essential when analyzed individually; however, we observed that the greater the number of atoms present in a structure, the greater its volume and topological polar surface area (TPSA), which are both characteristics that are essential for good oral absorption of the medication in the body, consequently obeying the Lipinski rule. On the other hand, it is not the only relevant characteristic for the prediction of the values of inhibitory activity and must be corroborated with the other characteristics provided by the statistical analysis [15]. Figure S2 represents the PCA analysis for the selected molecules. It correlates its characteristics with the inhibitory activity; the compounds with the lowest activity are in red, and those with the best activity are in blue. Molecule **11** is displaced from the others because it presents values of hydrogen donors equal to 3, which is different from the others in the selected training group that present values of 0 and 1, showing statistically a decrease in the pIC_50_ values of the structures. 

In addition, the values of the number of atoms provide a better forecast: molecule **11** (37 atoms) shows one of the lowest values for ATM, as well as **15, 18, 19,** and **20**. It is observed that for the most active molecules, the ATM characteristics are relevant to the value of inhibitory activity, shifting them to the most active side. All the most active structures have four aromatic groups, except for **molecule 4** which has only 2; however, its inhibitory activity is accentuated by the number of atoms in its structure (50).

In parallel, it must be understood that the predicted activity depends on the correlation between the four selected characteristics and their relative weight, and that the objective of the preliminary QSAR analysis is to investigate the most relevant characteristics among the data presented in the sampling of the structures already reported with inhibitory activity that are selective for COX-2. Figure S3 shows the analysis of HCA for the selected inhibitors. The HCA analysis gathers in hierarchical groups by similarity; the most active are represented in blue, and the least active ones are in red. It is observed that the data from the cluster followed the data obtained previously via QSAR and principal component analysis (PCA) analyses.

**Figure 3:**
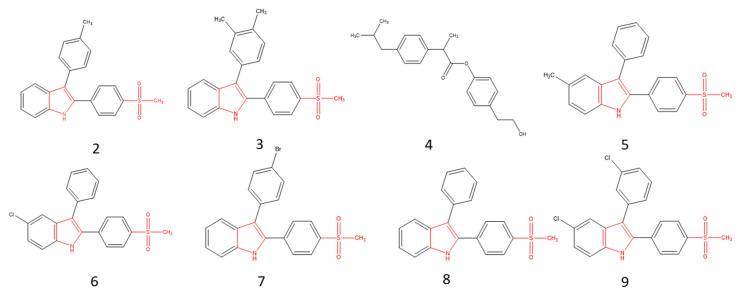
The most active molecules.

The tree-like dendrogram is seeking the structural similarities and the response to the inhibitory activity. It is noticed that **2** to **9** have four aromatic groups, with the exception of **4**, which has only two, but its activity is enhanced by the number of atoms of the structure. Figure 3 shows the structure of the eight most active molecules. The common group observed in these inhibitors is pyrrole, in addition to the methylsulfone group, with the exception of **4**. The presence of the methylsulfone group resembles the structure of rofecoxib and differs from the structures of the other coxibs (celecoxib and etoricoxibe), which have a sulfonamide functional group that is responsible for their toxicological characteristics. Pyrrole gives the appropriate lipophilic character to the molecule, which can help the molecule enter the COX-2 active channel [16].

Table 2 shows the regression data of the descriptors used to verify the best model for predicting the inhibitory activity. A combination has been used to evaluate the statistical parameters and select the parametric prediction equation according to the best fit. It is observed that the physical-chemical parameters ATM, ACC, and ARO are significant for the final calculated pIC_50_ values. The best statistical parameters were obtained for the parametric tri and tetra models with R^2^ values of 0.9599 and 0.9617, and variance ratios of 62.6373 and 46.2719, respectively. It is emphasized that the greater the number of equated scores, the greater the quality of the predictor model, although the values are statistically close [10].

Note that the parametric tetra prediction values were better adjusted with a correlation R = 0.9617 and standard error of estimate (SEE) = 0.2238, with a notable predictive capacity. Such alignment can be compared with the residual values found during the validation step of the equation, with Δ4 differences close to 0.2, which demonstrates the ability to predict the values of inhibitory activity. Table 3 analyzes the predicted models for the molecules, allowing inference of the difference (Δ = residual) between the pIC_50_ values found in an experimental way and the statistical prediction values for a defined parametric model and the equation was determined considering the highest statistical correlation.

The best results in question were for **10** (Δ4 = 0.0075) and **12** (Δ4 = 0.0020) inhibitors, although **2** (Δ4 = 0.4764), **16** (Δ4 = −0.2779), and **18** (Δ4 = −0.2625) present residues greater than 0.2 in relation to the experimental data obtained. The margin of error (SEE = 0.2238) allows us to infer that the two may be within the desired perspective of residue, mainly, less than 1. The internal validation set demonstrated the detection of anomalous samples, which were excluded from the test set because they reduced the statistical correlation of the applied parametric method, with residues greater than 0.4, increasing the estimated error and deviating the correlation of the predicted values with the experimental data, which is justified by its exclusion from the test set initially. However, the reinclusion of these aims to determine the predictive capacity of the model, and results with residuals less than 1 are significant.

**Figure 4:**
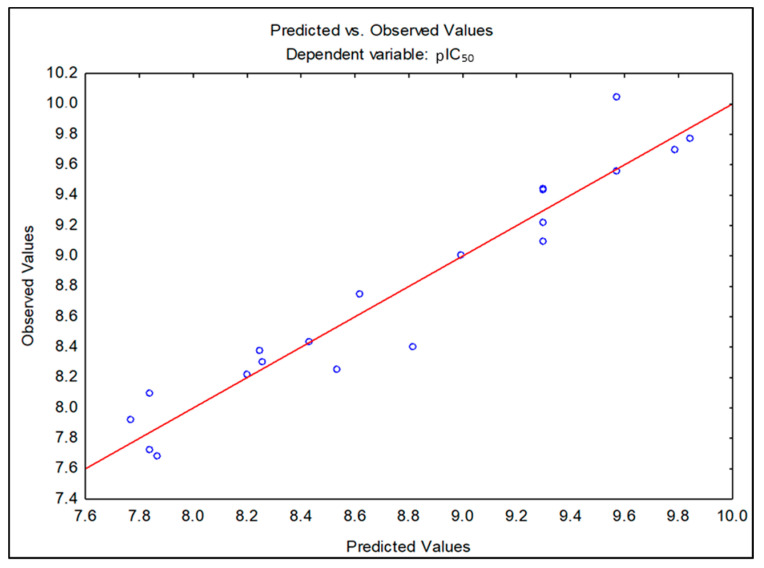
Linear correlation graph of the tetra-parametric model.

Figure 4 shows the projection of the data obtained in relation to the linear regression obtained, with a line adjustment of R^2^ = 0.9617 of the tetra-parametric model, showing a good relationship between the experimental and predicted values.

Table 4 shows the predicted values for the external validation set, applying the equations according to Table 4. For the validation test, a set between 20% and 30% of the total of the original set were used in the predictive model in order to prove its robustness [10]. It is observed that the values have good predictive quality for the molecules selected as an external set, with greater proximity for **26**, **27**, and **30**, with residues close to 0.1. This shows that the model has a significant correlation between the descriptors used.

### 2.2. Virtual Screening and Analysis of Pharmacokinetic and Toxicological Properties

After selecting the best inhibitors, these were used on the Protox II and Molinspiration servers to select the reduction filters; the compounds were directed to the ZincPharmer database through the Pharmit web server (http://pharmit.csb.pitt.edu/search.html) shown in Table S2, with the maximum and minimum values being selected in order to limit the promising structures to those within the pre-applied characteristics through the QSAR analysis, with a maximum limit of 2000 structures to be selected. The filters are applied on the online server, as well as the pharmacophore coordinates (below) elucidated for possible data reproduction and comparison with statistical analysis. 

The pharmacophore structure obtained through the PharmaGist server is demonstrated, aligning the similarities of the twenty selected molecules (Table 5). It is observed that the characteristics of the pharmacophore follow the data obtained through statistical analysis, presenting two aromatic groups, two hydrophilic groups, and hydrogen receptors. Such pharmacophore characteristics are essential when compared to the central process molecule, which has two ARO groups and four ACC groups, allowing the tracking of molecules with physical and chemical characteristics closer to rofecoxib. 

On the other hand, in studies by Chakraborty, Sengupta, and Roy (2004) [17], linear multiple regression (RML) analyses were used to deduce statistically acceptable equations. The variation ratios were 0.675 for COX-1 and 0.842 for COX-2, observing three important pharmacophores groups: methyl sulfonyl portion, central phenyl ring, and terminal phenyl ring. These are relevant when compared to their affinity with the lipophilic channel present in the active sites of the enzymes, corroborating with the data obtained in the present study.

After the application of the reduction and selection filters of the 2000 compounds, they were submitted to similarity to Tanimoto to find out which ones are closer to the characteristics of the pivot molecule used in the process (Rofecoxib). The fifty eight (58) molecules were obtained with a Tanimoto index greater than 0.35 (see Table 6), which is a value that is considered reasonable for the application of toxicological and pharmacological prediction studies in silico, with three promising molecules being selected during these tests as reported below, subsequently applying the molecular coupling and molecular dynamics tests [18]. 

Table 7 shows the best results of the toxicological tests applied to the three inhibitors selected through the Tanimoto index and tests performed through the online server PreADMET (https://preadmet.bmdrc.kr/adme/) in order to screen those who present better absorption, distribution, and metabolism values besides limiting the possibilities of mutagenicity through toxicological tests. It is observed that molecules showed high LD_50_ values, with the exception of **Z-627**, but it presents good values for the absorption and distribution tests, contributing to its selection in the molecular docking tests. Carcinogenicity tests for rats and mice demonstrate a possibility of mutation for all the selected inhibitors; however, when compared to the control compound, it also observed this important side effect, and accordingly, it did not prevent the selection of these molecules. At the same time, the Ames test was used as a cut-off parameter between the most promising and those that would be excluded from the subsequent steps, where those that showed a positive result were eliminated from the process. This test assesses the possibility of mutagenicity of chemical compounds in media with a low histidine concentration, which allows the strains of *Salmonella typhimurium* to change and return to a prototypical state, which directly influences the carcinogenic response.

The pharmacokinetic data for distribution are shown in Table 8. The plasma protein binding values (PPB) refer to the degree of binding of the inhibitors with the proteins present in the blood and C_brain_/C_blood_ represents the permeability of the blood–brain barrier. Compounds with C_brain_/C_blood_ values less than 1 do not have activity on the central nervous system (CNS).

It is observed that **Z-964** shows 100% of binding with the plasma proteins, inferring the possibility of its bioaccumulation and a consequent increase in its half-life within the organism, since the unbound portion is metabolized, consequently is excreted, and the bound part is slowly released in order to maintain the balance of the medium. In parallel, **Z-627** showed an 85% binding, indicating that 15% of the fraction will not be bound, which increases the efficiency of diffusion and penetration into the cell membranes [19]. All selected compounds have no activity on the central nervous system, as they show values below one. In silico values for absorption are shown in Table 9.

The selected drug candidates showed high values for intestinal absorption (HIA> 94%), being one of the most important absorption, distribution, metabolism and excretion (ADME) properties [20]. The drug molecules are transported from the gastro-enteric tract to the blood circle and permeate the gastro-enteric membrane by various mechanisms, and among them, the activity of the P-glycoprotein must be taken into account. This P-glycoprotein is a common transporter in the intestinal penetration of drugs, inferring in the hypothesis that the inhibitors **Z-964** and **Z-814**, because they present an in vitro inhibition of P-gp, decrease the efflux process through the passive permeability of the inhibitors, which is mediated by this protein. However, they have considerable absorption values when compared with those of the other molecules screened in this study.

The P_MDKC_ permeability value is significant for the **Z-814** inhibitor (28.3061 nm/sec), being higher than for the control compound. Values greater than two indicate a significant medication efflux. The compound **Z-964** showed a low permeability MDCK (0.0517 nm/sec) and **Z-627** approached the ideal (1.4352 nm/sec) [21,22]. In parallel, the Caco-2 permeability assay measures the flow rate of a compound through Caco-2 cell monolayers to predict the in vitro drug absorption, where values greater than two present drug efflux. Inhibitors **Z-814** (PCaco-2 = 12.4185 nm/sec) and **Z-964** (PCaco-2 = 42.9100 nm/sec) have higher values than rofecoxib (PCaco-2 = 2.7291 nm/sec), with the **Z-627** inhibitor (PCaco-2 = 0.6460 nm/sec) having a lower value; however, it is not a P-gp inhibitor, which can significantly interfere with intestinal absorption [22,23].

Table 10 demonstrates the predicted data for the biological activity of the selected inhibitors and compares the results against the selected controls rofecoxib and celecoxib, which were obtained from the PASS server (http://www.pharmaexpert.ru/passonline/). Celecoxib is used in everyday clinical practice, being part of the set of external validation and molecular docking of this research. The three selected inhibitors showed Pa > Pi values, indicating the possibility of activity in relation to the reported biological activities, mainly in terms of anti-inflammatory responses. **Z-627** has the best values for anti-arthritic (Pa = 0.985) and anti-inflammatory (Pa = 0.852) activities higher than controls (**Z-627** = 0.852, rofecoxib = 0.828, celecoxib = 0.663). 

All the candidate inhibitors have the possibility of activity against COX-2, although it is below the value of the reference compounds. Nonetheless, it serves as a reference for possible activities that they may present during the in vivo tests to be performed. In addition, a prediction of adverse effects that they may have on the organisms was performed, verifying that all of them present a propensity of activity similar to the other selected control compounds (celecoxib and rofecoxib) in the case of extrapyramidal effects. However, they have a lower propensity for the emergence of gastrointestinal problems, such as ulcers, which is the main focus in the development of new selective anti-inflammatory drugs, and the **Z-964** structure did not present the possibility of presenting such an adverse effect. Table 11 shows the physical–chemical data of the selected inhibitors.

Knowing the possibility that the structures present adverse cardiotoxic risks, the results were compared with the molecule still marketed in celecoxib, now considering its performance. However, it is observed that it presents risks of myocardial infarction and heart failure, which are analyzed through the Metatox (http://way2drug.com/mg2/gen_meta_all.php) and the hERG study (Human ether-a-go-go), through the PreADMET server, which refers to the blocking of the potassium channel, and that may cause cardiac collateral damage; see Table 11 below.

In view of the foregoing, this fact still did not allow its withdrawal from the market, as follow-up and adequate dosage reduce side effects and toxicological risks, which is a valid narrative for every drug currently sold; therefore, its cost–benefit must be evaluated. For now, it is seen that the molecules present a risk similar to or below the molecule withdrawn from the market (rofecoxb) and that which is still on the market (celecoxib), which does not preclude the possibility of being evaluated as candidates for specific COX-2 inhibitors. It is observed that the structures Z-814 and Z-627 present low and medium hERG risk, respectively, being better or equal to the molecules already commercialized, which makes their application as candidates for inhibitors of cyclooxygenase-2 possible. The Z-964 structure remains in the study due to the good results of bioavailability, in order to evaluate its experimental response in another study, as well as the others.

All candidate inhibitors present physical and chemical data within the acceptable range, showing no violation (Nv) or violating Lipinski’s rule of five. This rule says that drugs with good oral bioavailability must obey four physicochemical parameters: molecular weight (MW) ≤ 500 g/mol, octanol/water partition coefficient (log *P*) ≤ 5, the number of hydrogen-bond donor groups (nHD) ≤ 5, and the number of hydrogen-bond acceptor groups (nHA) ≤ 10, see Table 12. 

**Figure 5:**
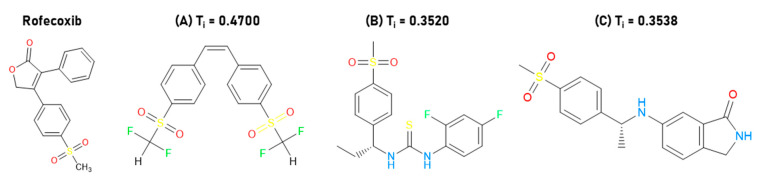
Selected inhibitors with Tanimoto index. (**A**) **Z-814**; (**B**) **Z-964**; (**C**) **Z-627**.

Table 12 shows that they have good absorption or permeability [24]. The pIC_50_ values (nM) were predicted for the selected molecules, see Table 13, according to the equations of Table 4, demonstrating acceptable values. The three selected inhibitors with Tanimoto index are shown in Figure 5.

### 2.3. Molecular Docking

**Figure 6:**
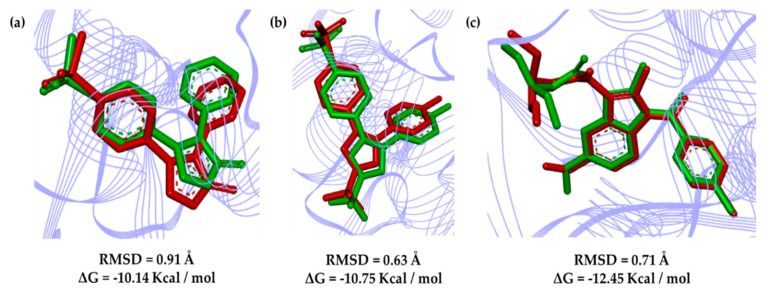
Overlapping poses of the crystallographic complexes (in green) with calculated poses (in red): (**a**) rofecoxib (**RCX**) for *Homo sapiens* (PDB 5KIR), (**b**) celecoxib (**CEL**) for *Mus musculus* (PDB 3LN1) and (**c**) indomethacin (**IMS**) *Ovis aries* (PDB 2OYE).

Figure 6 shows the poses calculated in relation to the deposited PDB complexes, with the deviation of the mean square root (RMSD) calculated at 0.91 Å for rofecoxib (RCX; 5KIR PDB code), 0.63 Å for celecoxib (**CEL**; 3LN1 PDB code) and 0.71 Å for indomethacin (**IMS**; 2YOE PDB code). Such a methodology provides alignment values for a maximum of 2 Å for the study of molecular docking, and accordingly, it validates the protocols used [25,26].

**Figure 7:**
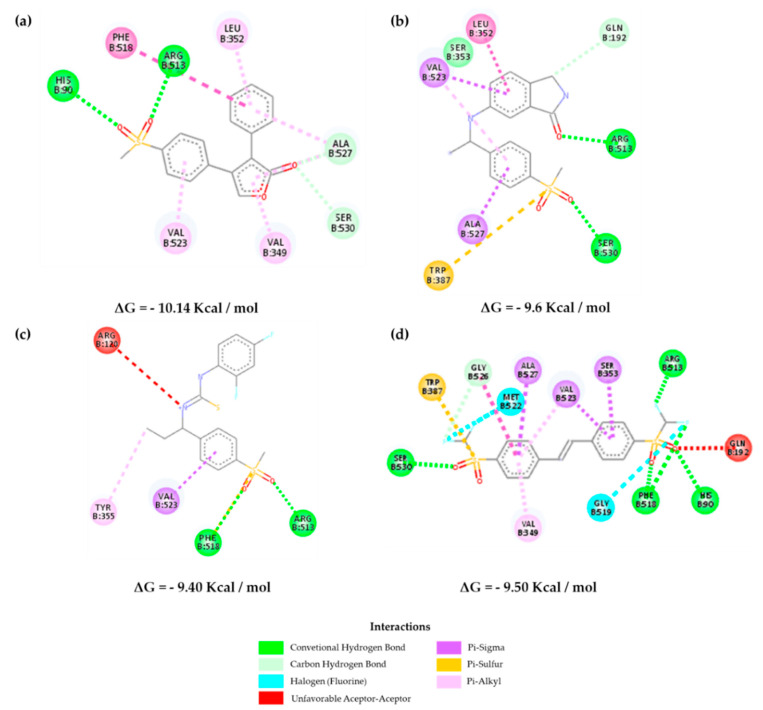
Interactions of the inhibitors (**a**) rofecoxib, (**b**) **Z-627**, (**c**) **Z-964,** and (**d) Z-814** with the active site of the structure of the Vioxx bound to the *Homo sapiens* cyclooxygenase-2 (COX-2, PDB 5KIR).

Figure 7 shows the interactions of the selected inhibitors with the control RCX in *Homo sapiens*. It is known that COX-1 and COX-2 have practically identical tertiary structures; however, the main difference between both is the replacement of Ile^434^, His^513^, and Val^434^ residues in COX-1 by Val^434^, Arg^513^, and Val^523^ in COX-2, respectively. This allows an increase of approximately 25% of the active site that consists of a more accessible pocket with Arg^513^ as a fundamental bonding site [16,27]. Figure 7a shows the main interactions of rofecoxib, within the pocket that provides a selective inhibition.

It is observed that the selected molecules **Z-814**, **Z-964**, and **Z-627** present a similarity of interactions with the amino acids of the hydrophobic region of the β leaf, Ser^530^, and Val^523^ for the former, and Ser^530^, Phe^518^, Val^523^, and Leu^358^ for the latter (Figure 7 and Table S3). The lipophilic channel of the enzyme is also constrained by the presence of Tyr^355^ and Arg^513^ on the enzyme surface, with the additional hydrogen-bond interaction between the Ala^527^ and Val^523^ phenolic group and the oxygen sulfone atoms of the structures. 

On the other hand, in COX-2, there are some interactions that allow greater accessibility in the lipophilic channel in this isoform than in COX-1, which can be observed, indicating a greater ease of interaction with the active site of COX-2 via Phe^518^. This effect can also be translated by the negative Gibbs free energy required for the interaction to occur (Figure S6). The inhibitors selected may have an equivalent affinity in relation to the selected control compounds. The interaction with Ser^353^ in **Z-814** demonstrates the possibility of a binding activity associated with low IC_50_ values [28,29,30].

**Figure 8:**
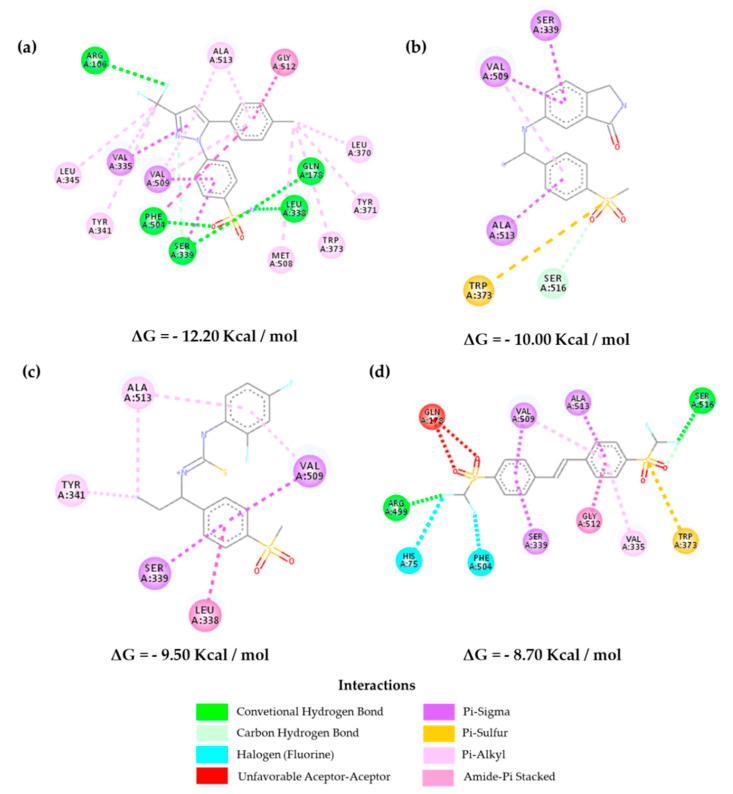
Interactions of inhibitors (**a**) celecoxib, (**b**) **Z-627**, (**c**) **Z-964**, and **(d) Z-814** with the active site of the *Mus musculus* COX-2 (PDB 3LN1).

In Figure 8 and Table S4, it is possible to verify the interactions of the selected inhibitors with the reference drug (**CEL**) against *Mus musculus*. Hydrophobic interactions are observed with Val^509^, Phe^504^, Gly^512^, Ser^339^, and Leu^338^, and hydrogen-bond interactions are observed with Gln^178^, Phe^504^, and Ser^339^ for **CEL**. In parallel, we can observe the interactions for the selected inhibitors, where **Z-627** shows interactions with the hydrophobic residues Ser^339^ and Val^509^ as well as the control, and in addition, it presents a hydrogen-bond with Ala^513^, showing selectivity [31,32].

On the other hand, the molecule **Z-964** shows greater interactions with Ala^513^, Ser^339^, and Val^509^ in the lipophilic region present in the β-leaf of the enzyme and the hydrophilic residue Leu^338^, which links to the sulfonic group of both inhibitors (sulfonamide for **CEL** and methylsulfone for **Z-964**). The **Z-814** molecule showed a lower affinity than the others, but it showed relative selectivity when it comes to the amino acid residues that are part of the interactions (Ser^339^, Val^509^, and Phe^504^ (fluorine)) in the hydrophilic region of the molecule, which allows for interactivity in parallel with the CEL molecule. Furthermore, the data corroborate the QSAR analyses carried out when dealing with the connections with hydrogen acceptors, which are mainly influenced by the electronegativity of the selected structures. These interactions have already been observed in other studies, corroborating with the affinity data shown in Figure S5, in which **Z-967** shows an energy of 10.00 kcal/mol and **Z-964** shows an energy of 9.50 kcal/mol. These data are considered the most important ones [30,31].

Docking studies corroborate the preliminary QSAR results, as they consider that the presence of aromatic groups can influence the inhibitory activity of such molecules; nevertheless, chemical changes are necessary in order to decrease in the cytotoxic effect of the inhibitors when compared with the reference drugs, such as the replacement of the sulfonamide by a methylsulfone group (rofecoxib analogs) [28]. The QSAR analysis demonstrates a structure–activity relationship, as is the case with the characteristics **ARO, ACC**, and **DONN**, being closely linked with the possibilities of their interaction with the active enzyme site. Lipophilicity deals with an intrinsic relationship of the possibility of permeation and good oral availability, which was previously reported with obedience to the rule of five by Lipinski (logP ≤ 5) interacting with the side pocket of the enzyme [30,31].

**Figure 9:**
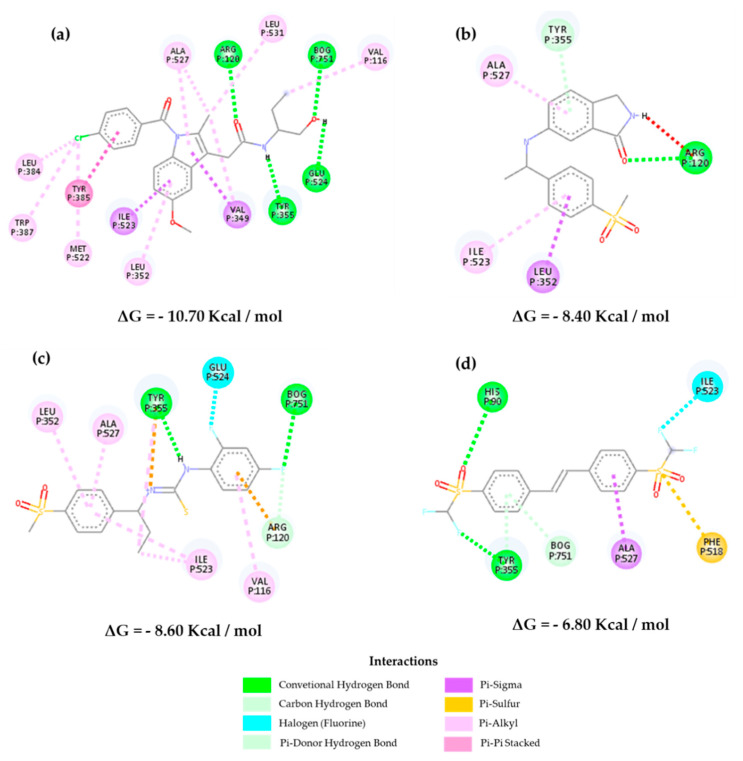
Interactions of the inhibitors (**a**) indomethacin, (**b**) **Z-627**, (**c**) **Z-964,** and (**d) Z-814** with the active site of the structure of the indomethacin complex to the *Ovis aries* COX-1 (PDB 2OYE).

**Figure 10:**
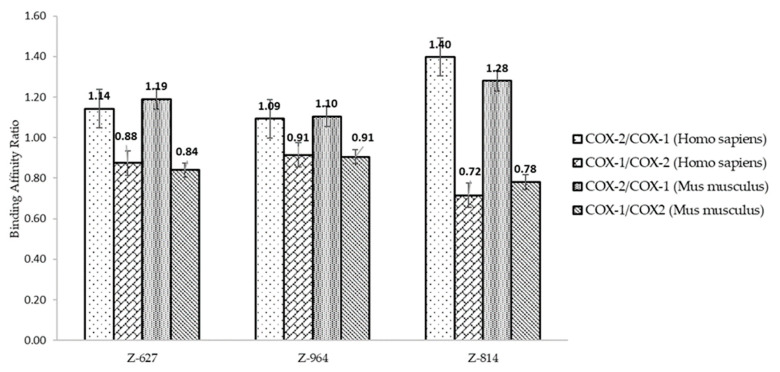
Binding affinity ratio of the selected structures.

The three structures were subjected to molecular coupling tests (Figure 9) to assess the possibility of being selective for COX-1 as well, which would determine the possibility of the appearance of undesirable adverse effects, such as gastrointestinal problems. They demonstrate a lower affinity possibility to the COX-2 enzyme as previously reported, with low bond energies (**Z-627** = −8.40 Kcal/mol, **Z-964** = −8.60 Kcal/mol, and **Z-814** = −6.80 Kcal/mol) compared to the selected control compounds and the indomethacin molecule (−10.70 Kcal/mol) deposited in the crystallographic structure of the PDB. The energy ratios (COX-2/COX-1 and COX-1/COX-2, as shown in Figure 10) were evaluated following an adaptation of the methodology adopted by Araújo and collaborators (2005) [7] that verified the influence of the most prescribed anti-inflammatory drugs of COX-2 on COX-1.

It is observed that the activity ratio on COX-1 is lower when compared to COX-2, suggesting that the structures will not be highly selective for isoform 1, emphasizing that all the NSAIDs already prescribed have a small selectivity for this, which decreases the possibility of side effects [7]. On the other hand, the perspective of the advent of adverse effects can be compared in parallel with the prediction of biological PASS activity (Table 10), indicating a probability of few gastrointestinal effects. Furthermore, it is noted that selectivity in relation to COX-2 is given by the substitution of valine for an isoleucine in COX-1 in position 523, which in this case interacts with the phenolic ring of the selected structures. In addition, most inhibitors selective for COX-2 suggest not having free carboxylate groups, which contributes to this low affinity to isoform 1, and the high affinity is expressed by the interaction as the amino acid residue Arg^120^ [33].

In this case, the **Z-627** structure presents this interaction relationship with the Arg^120^ residue in the pyrroline portion of the structure, showing a possible structural rigidity and suggesting a possibility of structural modification in order to further limit the relationship estimate. However, the addition of the methylene group in the residue from Ile^523^ indicates that interactions are restricted in access to the COX-1 side pocket, directly impacting the time-dependent competitive inhibition process in relation to COX-2 [34,35]. 

### 2.4. Structure–Activity Relationship of the Most Promising Molecules

The selected compounds (Figure 5) show a similar structure to that of the pivotal compound, having in their structures the methylsulfone group, showing no cytotoxic effect in relation to the sulfonamide group (**Z-627** and **Z-964**). Small substituents are the best, because they influence the volume of the molecule and possible van der Waals interactions with COX-2, which is a fact observed in docking studies. The introduction of fluorinated groups may show more significant activity. 

According to Hayashi et al. 2012 [36], substituted analogs by acceptable hydrogen-bond groups potentiate the inhibitor activity. On the other hand, substitutions in the isoindoline nucleus can contribute to the inhibitor–enzyme stabilization, further demonstrating the fundamental role that the electrostatic and dipole–dipole interactions can play [37,38].

At the same time, the endocyclic nitrogen atoms included in five- or six-membered cycles such as pyrrole, pyridine, and pyrimidine, among others, may produce an increase in selectivity. The five-amino group in the isoindoline ring may favor the inhibitory activity of **Z-627** and, moreover, possible hydrogen-bond interactions through the methylsulfone group. The inhibition mechanism depends on the prostaglandin biosynthesis by means of arachidonic acid (AA), estimating that AA fits into the channel cavity surrounded by amino acid residues with aromatic, aliphatic, and phenolic groups that establish several interactions. 

Therefore, competitive or selective inhibitors bind to Val^523^ in COX-2, interfering with the arachidonic acid cascade and preventing the peroxidase action, as well as the formation of prostaglandins or thromboxanes (pro-inflammatory eicosanoids). In parallel with the studies carried out by Hayashi et al. 2012 [36], the best inhibitors **Z-627** and **Z-814** have two hydrogen-bond donors, as well as low values of TPSA and MW. For the three selected inhibitors we proposed theoretical synthetic routes—Supplementary Materials Figures S9–S11.

### 2.5. Molecular Dynamics Results and Affinity Energy

**Figure 11:**
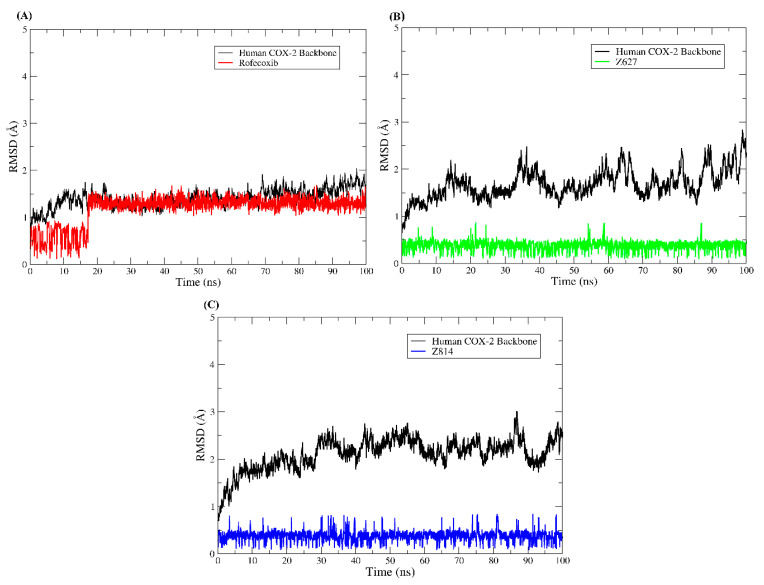
Root mean square deviations (RMSD) plot of complexes established with *Homo sapiens* COX-2. The protein backbone plot is colored black, but the ligand plots are colored in different ways. (**A**) RMSDs of the COX-2-rofecoxib system, (**B**) RMSDs of the COX-2-Z627 system, and (**C**) RMSDs of the COX-2-Z814 system.

**Figure 12:**
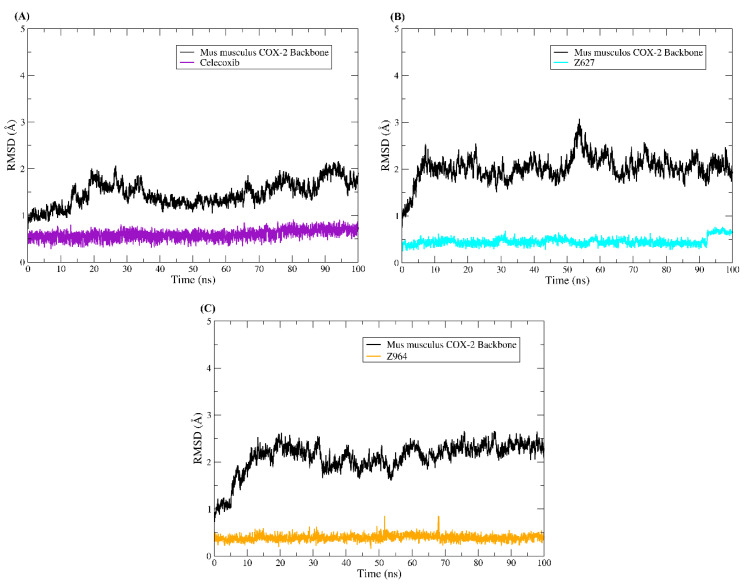
RMSD plot of complexes established with *Mus musculus* COX-2. The protein backbone plot is colored black, but the ligand plots are colored in different ways. (**A**) RMSDs of the COX-2-celecoxib system, (**B**) RMSDs of the COX-2-Z627 system, and (**C**) RMSDs of the COX-2-Z814 system.

The studies of molecular dynamics simulations were carried out to understand more deeply the modes of interaction of the selected compounds with the target proteins. The results obtained through molecular dynamics simulations have served as support for the detailed evaluation of conformations over time observed in drug–receptor complexes [39,40,41]. Thus, understanding that the dynamics and changes in the movement of a protein are closely related to its biological function allows us to understand that the observation of these phenomena is extremely important. In this way, we carried out the investigation of the protein structure during the 100 ns of molecular dynamics simulations using the methods of root mean square deviations (RMSD) and root mean square fluctuations (RMSF). To plot the RMSD of the ligands, all the heavy atoms of the molecules were used, while to plot the RMSD and RMSF of the protein backbone, the Cα carbon atoms were used. In Figure 11, the graphs of the compounds that were bound to COX-2 of *Homo sapiens* are plotted, while in Figure 12, the RMSD plot of the complexes established with COX-2 of *Mus musculus* is displayed.

Along the trajectories of MD simulations, COX-2 showed differences in the RMSDs of the complexes. The maximum Plator rising by the backbone RMSD was 3 Å, which was visualized in the COX-2-Z814 system, and the smallest fluctuation was observed in the COX-2-rofecoxib system. Despite the fluctuations displayed, this did not impair the interaction with the complexed ligands. It is important to note that the RMSD of the ligands showed low fluctuations and had a low RMSD value; this means that the ligands did not undergo drastic conformational changes after settling at the protein binding site.

Similar phenomena were observed for the complexes established between *Mus musculus* COX-2 and ligands. The backbone RMSD Plator was approximately 3 Å, and the ligands also remained in equilibrium throughout the 100 ns simulations, as observed in the RMSD graphs with small fluctuations.

**Figure 13:**
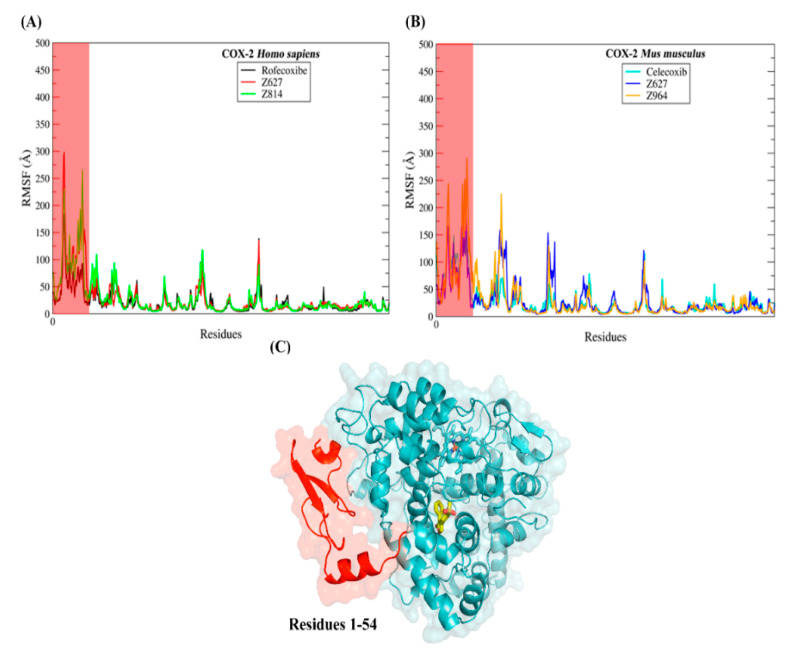
Root mean square fluctuations (RMSF) plot of the backbone of the proteins that established complexes with the compounds obtained by virtual screening. (**A**) RMSF for the COX-2 *Homo sapiens;* (**B**) RMSF for the COX-2 *Mus musculus.* (**C**) The region of the protein that has undergone the greatest fluctuations is highlighted in red.

The evaluations of the regions of the protein that obtained the greatest fluctuations along the trajectories of molecular dynamics were performed using the RMSF plot (see Figure 13). In general, the RMSF graphs showed a similar profile, even in the regions that suffered the greatest fluctuations. The greatest fluctuations were observed in the N-terminal portion of the protein.

This region is exposed to the solvent, being formed by alpha helices and beta leaves that are connected by long loop regions. Structurally, loops are the most flexible regions of the protein, so a region that exhibits many loops has a tendency to be flexible, as was observed in the RMSF plots displayed. Although this region is close to the active site of the ligands, its flexibility did not compromise the binding of the compounds, since all the ligands showed energy of favorable affinity with the protein, according to the molecular mechanics/generalized born surface area (MM-GBSA) results obtained. In addition, the fluctuation of this region did not affect the conformational stability of the ligand along the trajectory of molecular dynamics, as the RMSD graphics of the ligands demonstrated that they remained stable along the trajectories without showing drastic changes in the RMSD plot.

In addition to structural analysis of the protein and ligands using RMSD and RMSF, we also evaluated whether the compounds are capable of interacting favorably with molecular targets. For this, we use the MM-GBSA method. The results obtained are summarized in Table 14.

All ligands have been shown to be able to interact favorably with COX-2. The selected compounds showed great affinity with COX-2 when we compared their values of affinity energy with the value obtained for the positive control of protein in the human body and Mus musculus. In the system established with human COX-2, compounds **Z-814** (ΔG_bind_ = −48.15 Kcal/mol) and **Z-627** (ΔG_bind_ = −45.51 Kcal/mol) showed binding affinity values similar to that obtained for rofecoxib (ΔG_bind_ = −42.76 Kcal/mol), which was the positive control. 

Rofecoxib interacted through hydrogen bonds with the Arg^513^ and His^90^ residues, with an affinity energy of −2.14 and −1.82 Kcal/mol. Ligand **Z–814** established hydrogen bonds with His^90^ and Tyr^385^ with energy values of −1.53 and −1.48 Kcal/mol, while **Z–627** remained interacting with Phe^518^ and Ile^517^ with affinity values of −1.87 and −1.92 Kcal/mol. With the Mus musculus protein, the selected ligands, **Z–627** (ΔG_bind_ = −41.63 Kcal/mol) and **Z–964** (ΔG_bind_ = −44.27 Kcal/mol), also showed affinity values close to celecoxib (ΔG_bind_ = −47.78 Kcal/mol), which was used as a positive control. Celecoxib interacted with Phe^504^ and Ser^339^ through hydrogen bonds with affinity values of −1.42 and −1.95 Kcal/mol. Ligand **Z–627** interacted with Arg^499^ with an affinity of −1.81 Kcal/mol, while **Z–964** interacted with Phe^504^ with an affinity value of −1.68 Kcal/mol. The affinity energy values obtained with the MM-GBSA method, for the compounds selected by QSAR, were promising. This demonstrates that the selected substances can be considered as promising COX-2 inhibitors.

## 3. Materials and Methods

### 3.1. Selection of COX-2 Inhibitors

The molecule considered pivotal in the process was 4- (4-methylsulfonylphenyl)-3-phenyl-5*H*-furan-2-one (rofecoxib), which is known commercially as Vioxx^®^. It was taken from the BindingDB database (The Binding Database, https://www.bindingdb.org/bind/index.jsp) alongside twenty-four more molecules (Supplementary Material, Figure S7) to study the anti-inflammatory activity against COX-2 according to the literature data, following an increasing criterion of inhibitory activity, or IC_50_ (Table 15). The molecules were aligned using the Discovery Studio^®^ v. 4.0 program [42] for input on PharmaGist Web Server15 (http://bioinfo3d.cs.tau.ac.il/pharma/index.html) [43].

### 3.2. Optimization of Molecular Structures and Determination of Pharmacophore Characteristics

The selected inhibitors were pre-optimized by means of Molecular Mechanics (MM+), followed by calculations of Austin Model 1 (AM1) and PM3 in the Hyper Chem 7^®^ program (Table 2), with the lowest energy value used as a parameter of choosing the best model to carry out the construction of the pharmacophore hypothesis. Subsequently, the input was made to the PharmaGist Web Server 15 to determine the following characteristics: atoms (ATM), spatial characteristics (SF), characteristics (F), aromatic (ARO), hydrophobic (HYD), acceptor (ACC), and donor of hydrogen (DONN). The initial set presented 25 molecules, which were aligned according to the similarity with the selected pivot molecule, allowing the generation of pharmacophore models with the aid of the Discovery Studio^®^ v. *4.0* program, following the methodology developed by us [10,12,14,57,58,59].

### 3.3. QSAR and PCA/HCA Studies

The inhibitory activity values were transformed into pIC_50_ (−log (IC_50_)) in order to reduce the inconsistencies of the data obtained in an experimental way and homogenize the dataset, following the adopted methodological proposal [10,57,59]. In parallel, the importance of each pharmacophore descriptor was attributed—atoms (ATM), spatial characteristics (SF), characteristics (F), aromatic (ARO), hydrophobic (HYD), acceptor (ACC) and hydrogen donor (DONN); these were used for prediction in order to assess notoriety regarding the response to the pIC_50_ value through the Pearson correlation (p), using the software Statistica 7.0^®^ and Minitab 19^®^, adapting the methodology adopted by Santos et al. 2015 and Ferreira et al. 2019 [12,59]. Pearson’s coefficient (Equation (1)) measures the degree of linearity between two variables, assuming a value between +1 and −1. If one variable tends to increase while the others decrease, the value is negative. On the other hand, if both increase, the coefficient is positive. Moreover, $\bar{x}$ is the sample mean for the first variable; *s_x_* is the standard deviation for the first sample; $\bar{y}$ is the sample mean for the second variable; *s_y_* is the standard deviation for the second sample; and n is the column length.

(1) $$\rho =\;\frac{\sum_{i=1}^{n}\left(x_{i}-\;\bar{x}\right)\left(y_{i}-\bar{y}\right)}{\left(n-1\right)s_{x}s_{y}}$$

The best pharmacophore descriptors were obtained considering the statistical quality relations of multiple linear regression (MLR), such as correlation coefficient (R), correlation coefficient squared (R^2^), explained variance (adjusted R^2^), standard error of estimate (SEE), and variance ratio (F), and they were transformed into parametric models for predicting the inhibitory activity at pIC_50_ values. The combinations were obtained using four parameters indicated by Pearson’s correlation without repetition [12,59], according to Equation (2), where C = number of combinations, p = model type (p ≠ 0 and p = 4), and n = number of variables (n = 4).

(2) $$\mathrm{Cp},n=\frac{n!}{p!\left(n-p\right)!}$$

For the prediction of the best model, in the internal validation stage, the random correlations between the descriptors and the inhibitory activity were measured to normalize the data obtained, applying the technique of detecting anomalous samples (outliers), in order to obtain a homogeneous set. This subset is considered as internal validation, for analysis of the prediction capacity of the selected model, comparing the data obtained during the two validations (internal and external; Figure S8). Principal component analysis (PCA) together with hierarchical cluster analysis (HCA) were applied in order to verify whether the model obtained corresponds to the degree of similarity, using Pearson’s squared distance as a measurement parameter in the latter [60,61]. For the respective analyzes, Minitab v. 19^®^ trial version was used.

### 3.4. Virtual Screening and Selection of Inhibitor Compounds

After selecting the best model via QSAR analysis, the selected molecules were superimposed to form a pharmacophore model. After inputing the pharmacophore, the search was performed within the ZINC database, selecting the 2000 most similar molecules, using the partition coefficient (log *P*), surface area (TPSA), number of atoms (Natoms), Molar Mass (MW), hydrogen acceptors (nHA), hydrogen donors (nHD), number of violations (Nv), number of revolutions (Nrotb), and volume, which were values as filters determined via Protox II servers (http://tox.charite.de/protox_II/) and molinspiration (https://www.molinspiration.com/cgi-bin/properties). The RMSD (Equation (3)) value was used as a reference parameter, which is the measure of the average distance between the atoms of the overlapping inhibitors, given in Angstroms, representing the quantitative similarity relationship between them. The lower the RMSD value, the better the model is compared to the target structure. $\delta_{i}^{2}$ is the distance between atom i of any reference structure or the average position of N equivalent atoms.

(3) $$\mathrm{RMSD}=\sqrt{\frac{1}{N}\overset{N}{\underset{i=1}{\sum }}\delta_{i}^{2}}$$

Then, the Tanimoto test was performed via the BindingDB server. The similarity was determined according to the chemotype of the compounds screened with the pivotal molecule of the selection process to reduce and optimize the selection of compounds for determining pharmacokinetic and toxicological characteristics, using a cut-off index of 0.3, applying Equation (4) below [22].

(4) $$J=\frac{M_{11}}{M_{01}+M_{10}+M_{11}}$$

where M_11_—total number of attributes where A and B have a value of 1; M_01_—total attributes where A is 0 and B is 1; M_10_—total attributes where A is 1 and B is 0; M_11_—total attributes where A and B have a value of 0.

### 3.5. Prediction of Toxicological and Pharmacokinetic Properties

Pharmacokinetic and toxicological studies were applied to inhibitors extracted from Pharmit via the ZincPharmer server. PreADMET v. 2.0 (https://preadmet.bmdrc.kr/) was used, which is an application based on a database on the web that is used for the prediction of ADME data (Absorption, Distribution, Metabolism, Excretion) with the following being selected: blood–brain barrier (BBB) penetration, in vitro permeability in Caco2 cells, human intestinal absorption (HIA), in vitro permeability of MDKC cells, in vitro P-glycoprotein inhibition, plasma protein binding (PPB), and Toxicological for Ames_Test, Carcinogenicity for Rats and Mice. The LD_50_ values were determined via Protox II servers (http://tox. charite.de/protox_II/) as well as toxicity class.

### 3.6. Prediction of Biological Activity of Selected Inhibitors

Activity predictions were made using the online PASS server (http://www.pharmaexpert.ru/passonline), which allows you to predict the biological effects of compounds based on their formula using multilevel atom neighbors (VMA) descriptors, suggesting that the inhibitor’s activity is expressed in terms of its chemical structure. Molecules with activities reported for anti-inflammatory and cyclooxygenase inhibitor effects were selected [25,61].

### 3.7. Molecular Docking

For this step, only the molecules with the best pharmacokinetic, toxicological, and biological parameters were selected for the study of molecular docking, in order to evaluate the interactions with selected inhibitors and the respective targets through the measurement of free energy interaction with amino acid residues and binding affinity. The crystallographic poses were extracted from the web serve Protein Data Bank (PDB; https://www.rcsb.org/) for *Homo sapiens* with COX-2 complexed with the inhibitor rofecoxib having the code PDB 5KIR with a resolution of 2.697 Å, *Mus musculus* with COX-2 complexed with celecoxib having the PDB code 3LN1 with 2.40 Å resolution, and *Ovis aries* with COX-1 complexed with indomethacin having the PDB code 2OYE with 2.85 Å resolution; all structures were elucidated through X-Ray diffraction analyses.

#### Docking Study with AutoDock 4.2/Vina 1.1.2 via Graphical Interface PyRx (Version 0.8.30)

The selected inhibitors and proteins were prepared with the aid of Discovery Studio^®^ 4.0 software, and the evaluation of the complexes with the ligand was evaluated using the AutoDock 4.2/Vina 1.1.2 software and the PyRx graphical interface version 0.8.30 (https://pyrx.sourceforge.io), with the standard exhaustiveness parameter of the software being the best conformation obtained through the analysis of the RMSD value. The validation protocol was based on the determination of the x, y, and z coordinates according to the average region of the active site; these values are observed in Table 16. The energy function score was used to evaluate the free binding energy (ΔG) of the interaction of the receptors with the ligands [25].

The calculation of binding affinity (∆G) was also performed in order to compare the actual data obtained and the values predicted in silico, which was the same methodology adopted by Santos et al., 2020 [14], according to Equation (5).

(5) $$\Delta G=-RT\ln K_{i}$$

where R (gas constant) is 1.987.10^−3^ kcal·mol^−1^·K^−1^, the temperature is 310 K for rofecoxib/celecoxib, and K_i_ is 310.10^−9^ M for rofecoxib and 340.10^−9^ M for celecoxib [28,32,52].

### 3.8. Molecular Dynamics Protocol

The initial structure for the system was obtained from molecular docking methods. The restrained electrostatic potential (RESP) protocol with the HF/6-31G* basis sets was applied to obtain the partial atomic charges of the atoms of each ligand [62,63,64,65]. The parameters of the ligand were constructed with the Antechamber module [66] using General Amber Force Field (GAFF) [67].

The amino acid protonation state was characterized using the PDB2PQR server [68]. The systems were built with the tLEaP module of the Amber 16 package [69,70,71]. The force field used to describe the protein in all simulations was ff14SB [72]. The protein–ligand system was solvated in an octahedron periodic box containing water molecules in the TIP3P model [73]. The partial charges were neutralized by adding counter-ions.

Energy minimization occurred in four stages. First, the water molecules and ions were optimized using 2000 cycles of the steepest descent and 3000 cycles of conjugate gradient. Then, the position of receptor-ligand hydrogen atoms was optimized using 4000 steps of the steepest descent algorithm and 3000 steps of the conjugate gradient. At the third stage, hydrogen atoms, water molecules, and ions were further optimized using 2500 steps of the steepest descent algorithm and 3500 steps of the conjugate gradient. Finally, all atoms were minimized using 3000 steps of the steepest descent algorithm and three steps of the conjugate gradient.

Molecular dynamics simulations were performed at a constant volume by heating the systems up to 298 K. This heating was performed in five steps for a duration of 1 ns. After 100 ns, production runs were performed for each system.

The Particle Mesh Ewald method [74] was used for the calculation of the electrostatic interactions, and the bonds involving hydrogen atoms were restricted with the SHAKE algorithm - Restriction algorithm used to ensure that the distance between points of mass is maintained [75]. The temperature control was performed with the Langevin thermostat [76] within a collision frequency of 2 ps^−1^.

#### 3.8.1. Affinity Energy Calculations

To estimate the binding affinity (ΔG_bind_), we used the molecular mechanics/generalized born surface area (MM-GBSA) methods [77,78,79,80]. The ΔG_bind_ was calculated according to the following equations:

ΔG_bind_ = ΔG_complex_ − ΔG_receptor_ − ΔG_ligand_ (6)

ΔG_bind_ = ΔH − TΔS ≈ ΔE_MM_ + ΔG_solv_ − TΔS (7)

ΔE_MM_ = ΔE_internal_ + ΔE_ele_ + ΔE_vdW_ (8)

ΔG_solv_ = ΔG_GB_ + ΔG_NP_. (9)

The free energy of bonding (ΔG_bind_) is the summation of the interaction energy of the gas phase among the protein–ligand (ΔE_MM_), desolvation free energy (ΔG_solv_), and system entropy (−TΔS). ΔE_MM_ is the result of the sum of internal energy (ΔE_internal_, sum of the energies of connection, angles and dihedral) electrostatic contributions (ΔE_ele_), and the van der Waals term (ΔE_vdW_). ΔG_solv_ is the sum of the polar (ΔG_GB_) and non-polar (ΔG_NP_) contributions. ΔG_SA_ was determined from the solvent accessible surface area (SASA) estimated by the linear combination of pairwise overlaps (LCPO) algorithm.

#### 3.8.2. Per-Residue Free Energy Decomposition Analysis

Per-residue free energy decomposition was decomposed using the approach of MM/GBSA according to the following equation [14,81,82]:

ΔG_MM-GBSA_ = ΔE_vdW_ + ΔE_elec_ + ΔE_pol_ + ΔE_np_. (10)

## 4. Conclusions

After the pharmacophore-based virtual screening, the QSAR analysis demonstrated a good line fit with R^2^ = 0.96 and an equation with four main prediction parameters for pIC_50_, ATM, ARO, ACC, and DON, where the ARO, ACC, and DON report the relationship with the three new and promising compounds selected and the pivot structure (rofecoxib). The development of the predetermined multiple linear regression model predetermined the pIC_50_ values for the selected compounds **Z-814** = 7.9484, **Z-627** = 9.3458, and **Z-964** = 9.5272. In database searches to evaluate possible applications that may have already been carried out, these substances are not used in specific biological activities (https://scifinder.cas.org/ and https://zinc.docking.org/).

The analyzes of toxicological prediction and bioavailability confirm the possibility of significant activity of the structures with a reduction of possible undesirable effects, of which **Z-627** was considered the most promising in view of all the tests applied via ADME analysis, without consequences for the CNS; this was corroborated with the main compounds selected. All selected compounds have the methyl sulfone group, unlike coxibs, which have the sulfonamide group. These three molecules do not present toxicological risks; they comply with the Lipinski rule of five, which provides for good oral availability, and PASS provides for a specific activity with a high probability of showing promising anti-inflammatory activity, in addition to dim side effects in relation to the compound’s selected controls. Molecular coupling tests demonstrate strong energy affinity with isoform 2 and low activity with isoform 1 through relationship analysis, which induces a possibility of minor side effects. Finally, zebrafish larvae should be analyzed to assess anti-inflammatory activity in the treatment of inflammatory disorders to confirm in silico results.

## Figures and Tables

**Table 1:** Properties of the screened training molecules obtained from the PharmaGist server.

Inhibitor	Characteristics					
	ATM ^a^	ARO ^b^	DONN ^c^	ACC ^d^	pIC_50_	IC_50_ (nM)
1 *	36	2	0	4	7.9208	12.00
**2**	45	4	1	2	10.0457	0.09
**3**	48	4	1	2	9.7695	0.17
**4**	50	2	1	3	9.6989	0.20
**5**	45	4	1	2	9.5528	0.28
**6**	42	4	1	2	9.4436	0.36
**7**	42	4	1	2	9.4317	0.37
**8**	42	4	1	2	9.2218	0.60
**9**	42	4	1	2	9.0969	0.80
**10**	42	3	1	3	9.0000	1.00
**11**	37	2	3	5	8.7447	1.80
**12**	44	3	1	5	8.4317	3.70
**13**	40	3	1	3	8.3979	4.00
**14**	42	3	0	4	8.3767	4.20
**15**	29	2	1	2	8.3000	5.00
**16**	41	3	1	4	8.2518	5.60
**17**	40	1	0	4	8.2218	6.00
**18**	36	1	0	4	8.0969	8.00
**19**	36	1	0	4	7.7212	19.00
**20**	33	2	0	3	7.6777	21.00
ATM	1.0000	0.5659	0.2027	−0.2280	0.7651	−
ARO		1.0000	0.3559	−0.6492	0.7358	−
DONN			1.0000	−0.0423	0.4743	−
ACC				1.0000	−0.6399	−

* Pivotal molecule. [a] Atoms; [b] Aromatics; [c] Donors; [d] Acceptors.

**Table 2:** Parametric models and regression analysis values (R: Correlation coefficient; R^2^: Correlation to square coefficient; R_A_^2^: Correlation coefficient to adjusted square. SEE: Standard estimated error; F: Variance ratio).

**Mono Parametric**						
**Eq.**	**Descriptors used**	**R**	**R^2^**	**R_A_^2^**	**SEE**	**F**
1	ATM	0.7651	0.5854	0.5623	0.4804	25.4183
2	ARO	0.7358	0.5414	0.5159	0.5053	21.2522
3	ACC	0.6399	0.4095	0.3767	0.5733	12.4871
4	DONN	0.4743	0.2249	0.1819	0.6569	5.2251
**Bi Parametric**						
**Eq.**	**Descriptors used**	**R**	**R^2^**	**R_A_^2^**	**SEE**	**F**
1	ATM + ACC	0.9022	0.8140	0.7921	0.3311	37.2030
2	ARO + ATM	0.8487	0.7203	0.6874	0.4060	21.8906
3	ATM + DONN	0.8316	0.6916	0.6554	0.4263	19.0684
4	ACC + DONN	0.7809	0.6099	0.5640	0.4795	13.2914
5	ARO + DONN	0.7701	0.5930	0.5452	0.4898	12.3893
6	ARO + ACC	0.7661	0.5869	0.5383	0.4934	12.0789
**Tri Parametric**						
**Eq.**	**Descriptors used**	**R**	**R^2^**	**R_A_^2^**	**SEE**	**F**
1	ATM + ACC + DONN	0.9599	0.9215	0.9068	0.2217	62.6376
2	ARO + ATM + ACC	0.9053	0.8197	0.7859	0.3360	24.2481
3	ARO + ACC + DONN	0.8207	0.6736	0.6125	0.4521	11.0113
**Tetra Parametric**						
**Eq.**	**Descriptors used**	**R**	**R^2^**	**R_A_^2^**	**SEE**	**F**
1	ATM + ACC + DONN + ARO	0.9617	0.9250	0.9050	0.2238	46.2719

**Table 3:** pIC_50_ values calculated using the prediction equations.

Molecule	pIC_50_	Mono		Bi		Tri		Tetra	
		Eq. (1)	Δ1	Eq. (2)	Δ2	Eq. (3)	Δ3	Eq. (4)	Δ4
1 *	7.9208	8.2562	−0.3354	8.0305	−0.1097	7.7961	0.1247	7.7670	0.1538
**2**	10.0457	9.2561	0.7896	9.5495	0.4962	9.5773	0.4684	9.5693	0.4764
**3**	9.7695	9.5894	0.1801	9.8339	−0.0644	9.832	−0.0625	9.8414	−0.0719
**4**	9.6989	9.8116	−0.1127	9.6906	0.0083	9.6680	0.0309	9.7855	−0.0866
**5**	9.5528	9.2561	0.2967	9.5495	0.0033	9.5773	−0.0245	9.5693	−0.0165
**6**	9.4436	8.9228	0.5208	9.2651	0.1785	9.3226	0.1210	9.2972	0.1464
**7**	9.4317	8.9228	0.5089	9.2651	0.1666	9.3226	0.1091	9.2972	0.1345
**8**	9.2218	8.9228	0.2990	9.2651	−0.0433	9.3226	−0.1008	9.2972	−0.0754
**9**	9.0969	8.9228	0.1741	9.2651	−0.1682	9.3226	−0.2257	9.2972	−0.2003
**10**	9.0000	8.9228	0.0772	8.9322	0.0678	8.9888	0.0112	8.9925	0.0075
**11**	8.7447	8.3673	0.3774	7.7924	0.9523	8.5957	0.1490	8.6154	0.1293
**12**	8.4317	9.1450	−0.7133	8.4560	−0.0243	8.4910	−0.0593	8.4297	0.0020
**13**	8.3979	8.7006	−0.3027	8.7426	−0.3447	8.8190	−0.4211	8.8111	−0.4132
**14**	8.3767	8.9228	−0.5461	8.5993	−0.2226	8.3055	0.0712	8.2438	0.1329
**15**	8.3000	7.4785	0.8215	8.0327	0.2673	8.2189	0.0811	8.2529	0.0471
**16**	8.2518	8.8117	−0.5599	8.5045	−0.2527	8.5701	−0.3183	8.5297	−0.2779
**17**	8.2218	8.7006	−0.4788	8.4097	−0.1879	8.1357	0.0861	8.1972	0.0246
**18**	8.0969	8.2562	−0.1593	8.0305	0.0664	7.7961	0.3008	7.8344	0.2625
**19**	7.7212	8.2562	−0.5350	8.0305	−0.3093	7.7961	−0.0749	7.8344	−0.1132
**20**	7.6777	7.9229	−0.2452	8.0790	−0.4013	7.8752	−0.1975	7.8670	−0.1893
**21** **^[a]^**	9.3010	9.0339	0.2671	9.0270	0.2740	8.7242	0.5768	8.7066	0.5944
**22** **^[a]^**	8.8508	8.9228	−0.0720	9.2651	−0.4143	9.3226	−0.4718	9.2972	−0.4464
**23** **^[a]^**	8.8239	9.3672	−0.5433	9.6443	−0.8204	9.3127	−0.4888	9.3508	−0.5269
**24** **^[a]^**	8.6990	8.8117	−0.1127	8.8374	−0.1384	9.2534	−0.5544	9.2110	−0.5120
**25** **^[a]^**	8.4815	9.0339	−0.5524	9.3599	−0.8784	9.0580	−0.5765	9.0787	−0.5972
**Prediction Equations**									
Mono	pIC_50_ = 4.2566 + (0.1111 × ATM)								
Bi	pIC_50_ = 5.9493 + (0.0948 ×ATM) + (−0.3329 × ACC)								
Tri	pIC_50_ = 6.0749 + (0.0849 × ATM) + (−0.3338 × ACC) + (0.3495 × DONN)								
Tetra	pIC_50_ = 6.1250 + (0.0907 × ATM) + (−0.3721 × ACC) + (0.3766 × DONN) + (−0.0674 × ARO)								

* Pivotal molecule; [a] internal validation.

**Table 4:** External validation set.

Molecule	pIC_50_	Mono		Bi		Tri		Tetra	
		Eq. (1)	Δ1	Eq. (2)	Δ2	Eq. (3)	Δ3	Eq. (4)	Δ4
**26**	9.7447	9.3672	0.3775	9.3114	0.4333	9.6779	0.0668	9.6645	0.0802
**27**	9.2676	8.8117	0.4559	8.8374	0.4302	9.2534	0.0142	9.2110	0.0566
**28**	8.8508	8.9228	−0.0720	9.2651	−0.4143	9.3226	−0.4718	9.2972	−0.4464
**29**	8.6198	9.7005	−1.0807	8.2642	0.3556	8.2479	0.3719	8.1390	0.4808
**30**	8.2757	8.3673	−0.0916	8.1253	0.1504	8.2305	0.0452	8.1669	0.1088
**31**	8.1549	7.8118	0.3431	8.3171	−0.1622	8.4736	−0.3187	8.5250	−0.3701
Celecoxib	9.2839	8.7006	0.5833	8.4097	0.8742	8.4852	0.7987	8.4390	0.8449

**Table 5:** Pharmacophore characteristics.

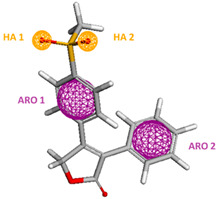	**Pharmacophore Characteristics**	**Coordinates**			
		**x**	**y**	**z**	**Radius**
	Hydrogen Acceptor (HA1)	15.30	−10.69	−1.79	0.50
	Hydrogen Acceptor (HA2)	17.52	−11.00	−2.88	0.50
	Aromatic (ARO1)	17.28	−7.30	−1.54	1.10
	Aromatic (ARO2)	21.57	−4.64	−1.16	1.10

**Table 6:** Similarity studies of molecules using the Tanimoto Index.

Zinc	Tanimoto Index (T_i_)		
	>0.25	>0.30	>0.35
Top Hits	1451	491	58

**Table 7:** Toxicological data of selected inhibitors.

Molecule	Toxicity				
	LD_50_ (mg/kg) ^[a]^	Toxicity Class ^[a]^	Ames_test	Carcino-Mouse	Carcino-Rat
Rofecoxib	4500	V	Mutagen	Negative	Negative
Celecoxib	1400	IV	Mutagen	Negative	Negative
**Z-814**	2000	IV	Non-mutagen	Negative	Negative
**Z-627**	61	III	Non-mutagen	Negative	Negative
**Z-964**	5000	V	Non-mutagen	Negative	Negative

**^[a]^** Protox (http://tox.charite.de/protox_II) Class I: fatal if swallowed (LD_50_ ≤ 5); Class II: fatal if swallowed (5 < LD_50_ ≤ 50); Class III: toxic if swallowed (50 < LD_50_ ≤ 300); Class IV: harmful if swallowed (300 < LD_50_ ≤ 2000); Class V: may be harmful if swallowed (2000 < LD_50_ ≤ 5000); Class VI: non-toxic (LD_50_ > 5000).

**Table 8:** Distribution data of selected inhibitors.

Inhibitor	Distribution	
	C_brain_/C_Blood_^[^^a^^]^	PPB (%) ^[^^b^^]^
Rofecoxib	0.0137	94.8036
Celecoxib	0.0272	91.0772
**Z-814**	0.9553	95.5607
**Z-627**	0.0344	85.3783
**Z-964**	0.4328	100.0000

**^[a^^]^** Permeability of the blood–brain barrier; **^[b^^]^** Plasma protein binding.

**Table 9:** Absorption data for selected inhibitors.

Inhibitor	Absorption			
	P_Caco-2_ (nm/sec) ^[^^a^^]^	HIA (%) ^[^^b^^]^	P_MDCK_ (nm/sec) ^[^^c^^]^	P-gp inhibition ^[^^d^^]^
**Rofecoxib**	2.7294	98.2248	11.2740	Non
**Celecoxib**	0.4994	96.6870	45.0486	Inhibitor
**Z-814**	12.4185	98.6322	28.3061	Inhibitor
**Z-627**	0.6460	94.4692	1.4352	Non
**Z-964**	42.9100	95.5974	0.0517	Inhibitor

**^[a^^]^** Cell permeability; **^[b^^]^** Human intestinal absorption; **^[c^^]^** Cell permeability Maden Darby Canine Kidney; **^[d^^]^** in vitro P-glycoprotein inhibition.

**Table 10:** Prediction of biological activity of selected inhibitors.

Inhibitor	Pa ^[a]^	Pi ^[b]^	Biological Activity	Pa ^[a]^	Pi ^[b]^	Adverse Effects
Rofecoxib	0.951	0.004	Antiarthritic	0.831	0.006	Extrapyramidal effect
	0.855	0.004	Non-Steroidal Anti-Inflammatory	0.640	0.006	Ulcer, peptic
	0.828	0.005	Anti-Inflammatory	0.531	0.040	Ulceration
	0.726	0.002	Cyclooxygenase-2 Inhibitor	0.385	0.024	Carcinogenic, group 1
Celecoxib	0.940	0.001	Cyclooxygenase Inhibitor	0.671	0.016	Pseudoporphyria
	0.936	0.001	Cyclooxygenase-2 Inhibitor	0.569	0.013	Ulcer, peptic
	0.809	0.007	Antiarthritic	0.498	0.024	Ulceration
	0.663	0.021	Anti-Inflammatory	0.385	0.134	Acidosis
**Z-627**	0.985	0.003	Antiarthritic	0.489	0.088	Extrapyramidal effect
	0.983	0.003	Antiosteoporotic	0.352	0.102	Visual acuity impairment
	0.852	0.005	Anti-Inflammatory	0.349	0.130	Fasciculation
	0.414	0.004	Cyclooxygenase Inhibitor	0.291	0.187	Interstitial nephritis
	0.192	0.016	Cyclooxygenase-2 Inhibitor	0.239	0.189	Ulcer, peptic
**Z-814**	0.821	0.006	Antiarthritic	0.761	0.015	Extrapyramidal effect
	0.569	0.024	Analgesic	0.747	0.037	Neutrophilic dermatoses
	0.527	0.049	Anti-Inflammatory	0.627	0.051	Hypercholesterolemic
	0.109	0.077	Cyclooxygenase Inhibitor	0.539	0.032	Adrenal contex hypoplasia
	0.084	0.065	Cyclooxygenase-2 Inhibitor	0.441	0.041	Ulcer
**Z-964**	0.689	0.007	Analgesic, Non-Opioid	0.428	0.106	Extrapyramidal effect
	0.688	0.018	Antiarthritic	0.383	0.083	Visual acuity impairment
	0.642	0.015	Analgesic	0.347	0.198	Nephrotic syndrome
	0.497	0.058	Anti-Inflammatory	0.313	0.166	Interstitial nephritis
	0.190	0.016	Cyclooxygenase-2 Inhibitor	0.309	0.207	Nephritis

**^[a^^]^** Pa = Possibility of activity; **^[b^^]^** Pi = Possibility of inactivity.

**Table 11:** Cardiotoxic effects of selected molecules.

Inhibitor	Pa ^[a]^	Pi ^[b]^	Adverse Effect ^[c]^	hERG ^[d]^
Rofecoxib	0.561	0.031	Cardiac Failure	Medium Risk
	0.547	0.036	Myocardial Infarction	
	0.345	0.305	Hepatotoxicity	
Celecoxib	0.528	0.043	Cardiac Failure	Medium Risk
	0.494	0.044	Myocardial Infarction	
**Z-814**	0.557	0.034	Myocardial Infarction	Low Risk
	0.464	0.074	Cardiac Failure	
**Z-627**	0.516	0.041	Myocardial Infarction	Medium Risk
	0.479	0.066	Cardiac Failure	
	0.710	0.096	Hepatotoxicity	
**Z-964**	0.503	0.054	Cardiac Failure	High Risk
	0.462	0.051	Myocardial infarction	

^[a]^ Pa = Possibility of activity; ^[b]^ Pi = Possibility of inactivity; ^[c]^ Metatox web; ^[d]^ PreADMET.

**Table 12:** Physicochemical data of the selected inhibitors.

Inhibitor	Properties							
	Milog *P* ^[a]^	TPSA ^[b]^	MW ^[c]^	nHA ^[d]^	nHD ^[e]^	Nv ^[f]^	Nrotb ^[g]^	Volume
Rofecoxib	0.71	60.45	314.36	4	0	0	3	264.79
Celecoxib	3.61	77.99	381.38	5	2	0	4	298.65
**Z-814**	3.35	68.28	408.39	4	0	0	6	299.11
**Z-627**	1.38	75.27	330.41	5	2	0	4	286.58
**Z-964**	2.03	58.20	384.47	4	2	0	7	316.16

^[a]^ Partition coefficient; ^[b]^ Topological Polar Surface Area; ^[c]^ Molecular Weight; ^[d]^ Number of Hydrogen Acceptors; ^[e]^ Number of Hydrogen Donors; ^[f]^ Number of violations; ^[g]^ Number of Rot bonds.

**Table 13:** Predicted pIC_50_ values of the selected inhibitors and controls.

Inhibitor	Molecular Properties				Parametric QSAR Models (pIC_50_ = −logIC_50_)			
	ATM ^[a]^	ARO ^[b]^	DONN ^[c]^	ACC ^[d]^	Mono	Bi	Tri	Tetra
Rofecoxib	36	2	0	4	8.2562	8.0305	7.7961	7.7670
Celecoxib	40	3	1	4	8.7006	8.4097	8.4852	8.4390
**Z-814**	38	2	0	4	8.4784	8.2201	7.9659	7.9484
**Z-627**	41	2	2	3	8.8117	8.8374	9.2534	9.3458
**Z-964**	43	2	2	3	9.0339	9.0270	9.4232	9.5272

^[a]^ Atoms; ^[b]^ Aromatics; ^[c]^ Donors; ^[d]^ Acceptors.

**Table 14:** Affinity energy of COX-2 ligands systems.

Organism	Ligand	ΔG_bind_ (Kcal/mol)
*Homo sapiens*	Rofecoxib	−48.15
	**Z814**	−45.51
	**Z627**	−42.76
*Mus musculus*	Celecoxib	−47.78
	**Z627**	−41.63
	**Z964**	−44.27

**Table 15:** Molecules selected in ascending order of IC_50_.

Inhibitor	Molecule (PDB Code)	IC_50_ (nM)	pIC_50_	References
1 *	Rofecoxib (BDBM22369)	12.000	7.9208	[44]
**2**	BDBM50272105	0.090	10.0457	[45]
**3**	BDBM50272097	0.170	9.7695	[45]
**4**	BDBM50365267	0.200	9.6989	[4]
**5**	BDBM50272130	0.280	9.5528	[45]
**6**	BDBM50272111	0.360	9.4436	[45]
**7**	BDBM50272124	0.370	9.4317	[45]
**8**	BDBM50272106	0.600	9.2218	[45]
**9**	BDBM50272092	0.800	9.0969	[45]
**10**	BDBM50049011	1.000	9.0000	[46]
**11**	BDBM50189988	1.800	8.7447	[47]
**12**	BDBM50057618	3.700	8.4317	[48]
**13**	BDBM50151689	4.000	8.3979	[17]
**14**	BDBM50103310	4.200	8.3767	[49]
**15**	BDBM13066	5.000	8.3000	[50]
**16**	BDBM50297680	5.600	8.2518	[51]
**17**	BDBM50026234	6.000	8.2218	[52]
**18**	BDBM50332773	8.000	8.0969	[52]
**19**	BDBM50332765	19.000	7.7212	[52]
**20**	BDBM50336012	21.000	7.6777	[53]
**21 ^a^**	BDBM50029613	0.500	9.3010	[54]
**22 ^a^**	BDBM50272113	1.410	8.8508	[45]
**23 ^a^**	BDBM50049030	1.500	8.8239	[46]
**24 ^a^**	BDBM50272109	2.000	8.6990	[45]
**25 ^a^**	BDBM50049013	3.300	8.4815	[46]
**26 ^b^**	BDBM50272128	0.180	9.7447	[45]
**27 ^b^**	BDBM50272090	0.540	9.2676	[45]
**28 ^b^**	BDBM50272113	1.410	8.8508	[45]
**29 ^b^**	BDBM50373566	2.400	8.6198	[55]
**30 ^b^**	BDBM50057606	5.300	8.2757	[48]
**31 ^b^**	BDBM50207446	7.000	8.1549	[56]
**Celecoxib ^b^**	BDBM11639	0.520	8.4390	[45]

* Pivot; a Internal validation; b External validation.

**Table 16:** Protocol data used in the validation of molecular docking.

Enzyme	Resolution	Inhibitor	Coordinates of the Grid Center	Grid Size (Points)
COX-2 (PDB code: 5KIR) *Homo sapiens*	2.400 Å	Rofecoxib	X = 23.214 Y = 41.353 Z = 3.517	36 x 26 y 28 z
COX-2 (PDB code: 3LN1) *Mus musculus*	2.697 Å	Celecoxib	X = 30.486 Y = −22.364 Z = -15.725	36 x 26 y 24 z
COX-1 (PDB code: 2OYE) *Ovis aries*	2.850 Å	Indomethacin (R)	X = 251.492 Y = 109.817 Z = −40.751	36 x 36 y 24 z

## Supplementary Materials

The following are available online, Table S1: Energy values of the optimized molecules, Table S2: Filters applied according to the properties of the selected molecules, Table S3: Distance of Interactions for the structures for the PDB 5KIR, Table S4: Distance of Interactions for the structures for the PDB 3LN1, Table S5: Distance of Interactions for the structures for the PDB 2OYE, Figure S1: Dendrogram representing clustering of pharmacophores, Figure S2: Analysis of the main components for the sorted molecules. Scores (a) and Loading Graph (b), Figure S3: Dendrogram of selected molecules. More active (blue) and less active ones (red), Figure S4: Binding affinity results of compounds, including Vioxx bound (COX-2 – Homo sapiens), Figure S5: Binding affinity results of compounds, including celecoxib (COX-2 Mus musculus), Figure S6: Binding affinity results of compounds, including Indomethacin (COX-1 Ovis aries), Figure S7: Structures used in the molecular modeling, Figure S8: Structures used in the external validation set, Figure S9: Theoretical synthetic route for the preparation of compound A (Z-814), Figure S10: Theoretical synthetic route for the preparation of compound B (Z-964), Figure S11: Theoretical synthetic route for the preparation of compound C (Z-627).

## Abbreviations

AA—arachidonic acid, ACC—acceptors, ADME—absorption, distribution, metabolism and excretion, ARO—aromatic, ATM—atoms, C_brain_/C_blood_—permeability of the brain barrier, CEL—celecoxib, COX-1—cyclooxygenase 1, COX-2—cyclooxygenase 2, DONN—donors, HCA—hierarchal components analysis, HIA—human intestinal absorption, IC_50_—inhibitory concentration, MDPI—Multidisciplinary Digital Publishing Institute, DOAJ—directory of open access journals, MilogP—partition coefficient, MW—molar weight, Natoms—number of atoms, nHA—number of hydrogen acceptors, nHD—number of hydrogen donors, Nrotb—number of rot bonds, Nv—number of violations, p—Pearson value, PCA—principal components analysis, Pcaco2—cell permeability, PDB—Protein Data Bank, P-gp inhibition—*In vitro* P-glecoprotein inhibition, P_MDKC_—cell permeability Maden Darby canine kidney, PPB—plasma protein binding, QSAR—quantitative structure–activity relationships, RCX—rofecoxib, RMSD—deviation of the mean square root, T_i_—Tanimoto Index, TPSA—topological polar surface area, Z-627—ZINC 170592627, Z-814—ZINC 33332814, Z-964—ZINC 225723964.
