# Supplementary material for: Identification of Potential COX-2 Inhibitors for the Treatment of Inflammatory Diseases Using Molecular Modeling Approaches
Source: Molecules. 2020 Sep 12;25(18):4183. doi: 10.3390/molecules25184183 (PMC7570943; doi:10.3390/molecules25184183)
Supplement: Supplementary file 1 [file molecules-25-04183-s001.pdf]

molecules

Article

Identification of potential COX-2 inhibitors for the treatment of inflammatory diseases using molecular modeling approaches

Pedro H. F Araújo [1,2], Ryan S. Ramos [2], Jorddy N. da Cruz [2], Sebastião G. Silva [3], Elenilze F. B. Ferreira [1,2,4], Lúcio R. de Lima [2], Williams J. C. Macêdo [1,2,5], José M. Espejo-Román [6], Joaquín M. Campos [6] and Cleydson B. R. Santos [1,2,5*]

1 Graduate Program in Innovation Pharmaceutical, Federal University of Amapá, Macapá, 68903-419 Amapá, AP, Brazil; pedro.henrique.fauro@gmail.com (P.H.F.A.); elenilze@yahoo.com.br (E.F.B.F.); williamsmacedo@yahoo.com.br (W.J.C.M.)
2 Laboratory of Modeling and Computational Chemistry, Department of Biological and Health Sciences, Federal University of Amapá, 68902-280 Macapá, AP, Brazil; ryanquimico@hotmail.com (R.S.R.); jorddynevescruz@gmail.com (J.N.C.); luciorolima@gmail.com (L.R.L.)
3 Abaetetuba University Campus, Ramal Manoel de Abreu, s/n-Mutirão, Abaetetuba, 68440-000 Pará, Brazil; profsebastiaogs@gmail.com
4 Laboratory of Organic Chemistry and Biochemistry, University of State of Amapá, 68900-070 Macapá, AP, Brazil
5 Laboratory of Molecular Modeling and Simulation System, Federal Rural University of Amazônia, Rua João Pessoa, 121, Capanema, 68700-030 Pará, PA, Brazil
6 Department of Pharmaceutical Organic Chemistry, Faculty of Pharmacy, Biosanitary Institute of Granada (Ibs.GRANADA), Campus of Cartuja s/n, University of Granada, 18071 Granada, Spain; josemanuelespejo@correo.ugr.es (J.M.E.-R.); jmcampos@ugr.es (J.M.C.)

* Correspondence: breno@unifap.br

SUPPLEMENTARY MATERIAL

Table S1. Energy values of the optimized molecules.

Table S2. Filters applied according to the properties of the selected molecules.

Table S3. Distance of Interactions for the structures for the PDB 5KIR.

Table S4. Distance of Interactions for the structures for the PDB 3LN1.

Table S5. Distance of Interactions for the structures for the PDB 2OYE.

Figure S1. *Dendrogram* representing clustering of *pharmacophores*.

Figure S2. Analysis of the main components for the sorted molecules. Scores (a) and Loading Graph (b).

Figure S3. Dendrogram of selected molecules. More active (blue) and less active ones (red).

Figure S4. Binding affinity results of compounds, including *Vioxx* bound (COX-2 – *Homo sapiens*). (*Values calculated form experimentally determined constants (K_i). ΔG = -RTlnK_i, were R (gas constant): 1.987.10^{-3} kcal.mol^{-1}.K^{-1}, Temperature: 310 K for rofecoxib and K_i: 310.10^{-9} M for rofecoxib.

Figure S5. Binding affinity results of compounds, including celecoxib (COX-2 *Mus musculus*). (*Values calculated form experimentally determined constants (K_i). ΔG = -RTlnK_i, were R (gas constant): 1.987.10^{-3} kcal.mol^{-1}.K^{-1}, Temperature: 310 K for celecoxib and K_i: 340.10^{-9} M for celecoxib

Figure S6. Binding affinity results of compounds, including Indomethacin (COX-1 *Ovis aries*).

Figure S7. Structures used in the molecular modeling.

Figure S8. Structures used in the external validation set.

Figure S9. Theoretical synthetic route for the preparation of compound **A** (**Z-814**).

Figure S10. Theoretical synthetic route for the preparation of compound **B (Z-964).**

Figure S11. Theoretical synthetic route for the preparation of compound **C (Z-627)**.

Table S1. Energy values of the optimized molecules.

Inhibitor	MM+ Energy (kcal/mol)	Time (sec)	AM1 Energy (kcal/mol)	Time (sec)	PM3 Energy (kcal/mol)	Time (sec)
**1***	50.6616	7	-4002.8432	32	-4012.291	34
**2**	63.3959	15	-5010.2054	25	-5029.8099	29
**3**	64.5118	16	-5292.7400	47	-5313.1109	48
**4**	19.3267	21	-5234.0158	38	-5231.9443	69
**5**	62.1416	13	-5010.0199	28	-5029.6662	22
**6**	62.0095	5	-4711.9704	38	-4729.5554	45
**7**	63.3396	7	-4697.9212	40	-4713.0742	53
**8**	63.4045	13	-4727.6932	30	-4745.3685	46
**9**	61.5203	12	-4695.6800	53	-4712.3226	54
**10**	224.9933	15	-4486.8486	49	-4487.6080	52
**11**	37.9820	8	-3988.1281	67	-4021.2991	68
**12**	54.9290	7	-4644.8192	40	-4652.8166	45
**13**	48.3832	17	-4657.2812	88	-4682.9454	87
**14**	50.3026	10	-4449.3169	57	-4448.3095	36
**15**	16.5530	12	-4739.4996	64	-4755.6782	53
**16**	46.8177	15	-3995.9617	48	-4021.6441	61
**17**	26.1888	22	-4488.5847	68	-4517.3085	21
**18**	14.6265	18	-3612.9458	76	-3614.5168	55
**19**	14.6482	12	-3878.8186	93	-3879.6921	102
**20**	18.9320	10	-3883.6023	52	-3891.1701	78
Average	53.2333	12.75	-4510.4400	51.65	-4524.5100	52.9

**pivotal molecule*

Table S2. Filters applied according to the properties of the selected molecules.

Inhibitor	Properties								
	**milog*P*[a]**	**TPSA**	**Natoms**	**MW[a,b]**	**nHA**	**nHD**	**Nv[a]**	**Nrotb[a,b]**	**Volume[a]**
**1***	**0.71**	60.45[a]/68.82[b]	36	314.36	4[a,b]	0[a,b]	0	3	264.79
**2**	4.87	49.93[a]/58.31[b]	45	361.46	3[a]/2[b]	1[a]/1[b]	0	3	320.39
**3**	5.25	49.93[a]/58.31[b]	48	375.49	3[a]/2[b]	1[a]/1[b]	1	3	336.95
**4**	4.73	**46.53[a,b]**	**50**	326.44	3[a,b]	1[a]/0[b]	0	8	325.18
**5**	4.85	49.93[a]/58.31[b]	45	361.46	3[a]/**1[b]**	2[a]/1[b]	0	3	320.39
**6**	5.08	49.93[a]/58.31[b]	42	381.88	3[a]/2[b]	2[a]/1[b]	1	3	317.37
**7**	5.23	49.93[a]/58.31[b]	42	**425.33**	3[a]/2[b]	1[a,b]	1	3	321.72
**8**	4.42	49.93[a]/58.31[b]	42	347.43	3[a]/2[b]	1[a,b]	0	3	303.83
**9**	**5.73**	49.93[a]/58.31[b]	42	416.33	3[a]/2[b]	1[a,b]	1	3	330.90
**10**	4.33	60.16[a]/68.54[b]	42	394.31	3[a,b]	2[a]/0[b]	0	3	314.69
**11**	1.26	**80.77[a,b]**	37	304.30	6[a]/5[b]	1[a,b]	0	3	260.72
**12**	3.97	77.99[a]/111.66[b]	44	427.46	5[a]/7[b]	2[a]/0[b]	0	5	**333.34**
**13**	4.85	60.16[a]/68.54[b]	40	359.39	3[a]/5[b]	2[a]/0[b]	0	3	296.00
**14**	2.54	46.61[a]/54.99[b]	42	329.41	4[a,b]	0[a,b]	0	3	291.74
**15**	4.57	49.33[a,b]	**29**	**296.15**	3[a,b]	2[a]/0[b]	0	4	**238.73**
**16**	4.22	71.95[a]/80.33[b]	41	393.38	5[a]/**8[b]**	1[a]/0[b]	0	5	310.24
**17**	5.48	55.77[a,b]	40	411.20	4[a]/7[b]	1[a]/0[b]	1	5	310.78
**18**	4.59	**46.53[a]**/71.83[b]	36	366.78	3[a]/7[b]	1[a]/0[b]	0	5	283.15
**19**	4.59	**46.53[a]**/71.83[b]	36	366.78	3[a]/7[b]	1[a]/0[b]	0	5	283.15
**20**	5.51	46.53[a,b]	33	389.15	3[a]/6[a]	1[a]/0[b]	1	3	283.21
**Maximum**	**5.73**	**80.77**	**50**	**425.33**	**8**	**2**	**1**	**8**	**333.34**
**Minimum**	**0.71**	**46.53**	**29**	**296.15**	**1**	**0**	**0**	**3**	**238.73**

**Pivotal molecule*; [a] Molinspiration; [b] Protox-II

Table S3. Distance of Interactions for the structures for the PDB 5KIR

Structure	Interactions	Aminoacid	Distance (Å)
Rofecoxib	Convetional Hidrogen Bond	Arg523B	3.07
		His90B	2.64
	Pi-Pi Stacked	Phe518B	5.82
	Pi-Alkyl	Leu352B	5.44
		Val523B	3.81
		Val349B	4.42
	Convetional Hidrogen Bond	Arg523B	3.07
		His90B	2.64
Z-627	Pi-Sigma	Val523B	4.58; 3.53
		Ala527B	3.78
	Van der Waals	Ser353B	3.55
	Conventional Hydrogen Bond	Arg513B	3.16
		Ser530B	2.36
	Carbon Hydrogen Bond	Gln192B	3.50
	Pi-Sulfur	Trp387B	5.15
	Amide-Pi Stacked	Leu352B	3.55
Z-814	Conventional Hydrogen Bond	Ser530B	2.27
		Arg513B	2.02
		His90B	3.02
		Phe518B	2.92
	Carbon Hydrogen Bond	Gly526B	2.72
	Halogen (Fluorine)	Met522B	2.30
		Gly519B	3.59
	Unfavorable Aceptor-Aceptor	Gln192B	2.78
	Pi-Sigma	Ala527B	3.72
		Val523B	3.80
		Ser353B	3.42
	Pi-Sulfur	Trp387B	5.02
	Pi-Alkyl	Val349B	5.37
Z-964	Conventional Hydrogen Bond	Phe518B	3.05
		Arg513B	2.17
	Unfavorable Aceptor-Aceptor	Arg120B	5.10
	Pi-Sigma	Val523B	3.81
	Pi-Alkyl	Tyr355B	5.77

Table S4. Distance of Interactions for the structures for the PDB 3LN1

Structure	Interactions	Aminoacid	Distance (Å)
Celecoxib	Convetional Hidrogen Bond	Gln178A	2.64
		Arg106A	3.68
		Leu338A	2.78
		Ser339A	3.71
		Phe504A	5.76
	Pi-Sigma	Val335A	4.70
		Val509A	5.47
	Pi-Alkyl	Leu370A	4.62
		Tyr371A	4.83
		Trp373A	5.50
		Met508A	4.87
		Tyr341A	5.35
		Leu345A	5.30
		Ala513A	4.69
	Amide Pi-Stacked	Gly512A	3.87
Z-627	Carbon Hydrogen Bond	Ser516A	3.51
	Pi-Sigma	Val509A	3.93
		Ser339A	3.63
		Ala513A	3.99
	Pi-Sulfur	Trp373A	5.15
Z-964	Pi-Sigma	Val509A	3.85
		Ser339A	3.34
	Amide-Pi Stacked	Leu338A	4.04
	Alkyl	Ala513A	3.60
	Pi-Alkyl	Tyr341A	5.35
Z-814	Convetional Hydrogen Bond	Arg499A	3.05
		Ser516A	2.76
	Halogen (Flurine)	His75A	3.20
		Phe504A	2.99
	Unfavorable Aceptor-Aceptor	Gln178A	2.75
	Pi-Sigma	Val509A	3.78
		Ser339A	3.51
		Ala513A	3.64
	Pi-Sulfur	Trp373A	5.43
	Amide-Pi Stacked	Gly512A	4.01
	Pi-Alkyl	Val335A	5.44

Table S5. Distance of Interactions for the structures for the PDB 2OYE

Structure	Interactions	Aminoacid	Distance (Å)
Indomethacin	Conventional Hydrogen Bond	Arg120P	3.19
		Bog751P	2.54
		Glu524P	1.87
		Tyr355P	1.98
	Pi-Sigma	Val349P	3.74
		Ile523P	3.85
	Alkyl	Leu384P	3.73
		Trp387P	4.71
		Met522P	4.30
		Leu352P	4.87
		Ala527P	3.44
		Leu351P	4.09
		Val116P	3.75
	Pi-Pi T-shaped	Tyr385P	5.51
Z-627	Conventional Hydrogen Bond	Arg120P	2.61
	Pi-Alkyl	Ala527P	4.73
		Ile523P	5.06
	Pi-Donor Hydrogen Bond	Tyr355P	3.58
	Pi-Sigma	Leu352P	3.74
Z-964	Conventional Hydrogen Bond	Tyr355P	1.90
		Bog751P	2.49
	Carbon Hydrogen Bond	Arg120P	3.36
	Halogen (Fluorine)	Glu524P	3.64
	Pi-Alkyl	Ile523P	3.62
		Ala527P	4.55
		Leu352P	4.98
		Val116P	4.93
Z-814	Conventional Hydrogen Bond	His90P	2.93
		Tyr355P	2.53
	Halogen	Ile523P	3.55
	Pi-Donor Hydrogen Bond	Bog751P	3.55
	Pi-Sigma	Ala527P	3.87
	Pi-Sulfur	Phe518P	5.55

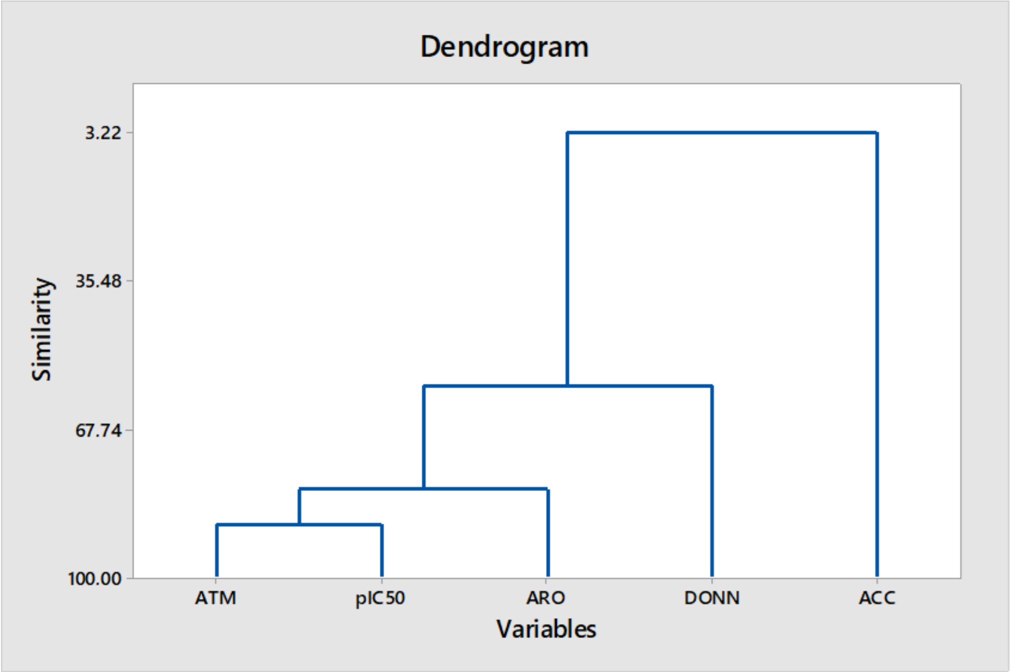

Figure S1. *Dendrogram* representing clustering of *pharmacophores*.

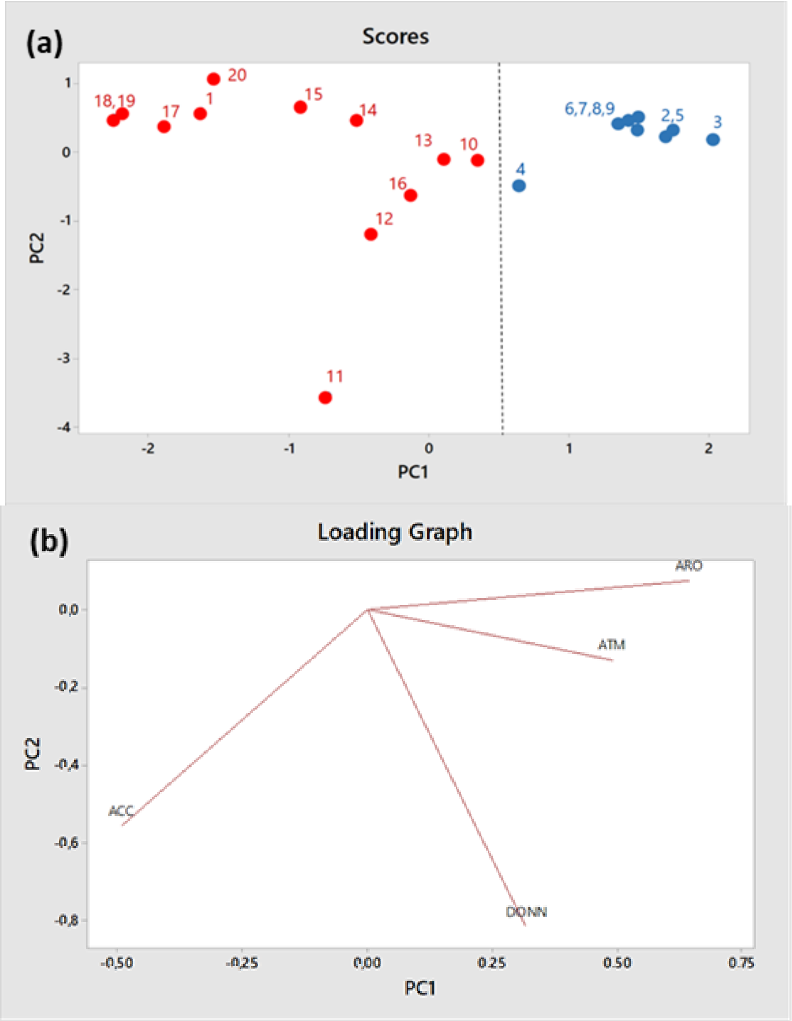

Figure S2. Analysis of the main components for the sorted molecules. Scores (a) and Loading Graph (b).

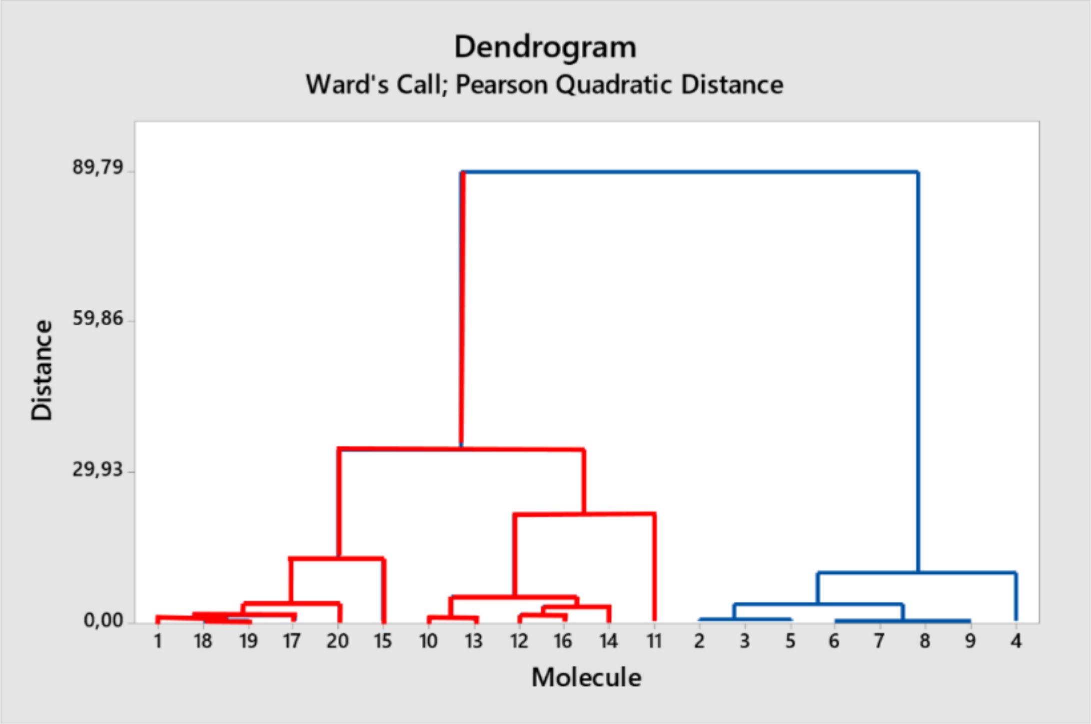

Figure S3. Dendrogram of selected molecules. More active (blue) and less active ones (red).

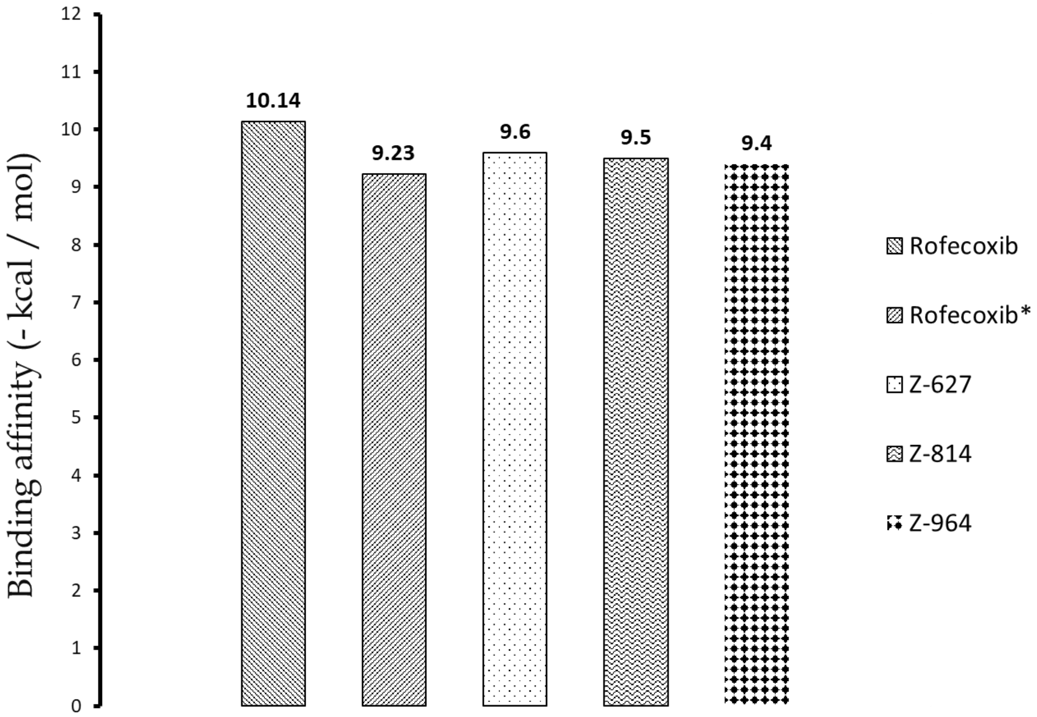

Figure S4. Binding affinity results of compounds, including *Vioxx* bound (COX-2 – *Homo sapiens*). (*Values calculated form experimentally determined constants (K_i). $\Delta G = -RT\ln K_i$, were R (gas constant): $1.987.10^{-3}$ kcal.mol^{-1}.K^{-1}, Temperature: 310 K for rofecoxib and K_i: 310.10^{-9} M for rofecoxib.

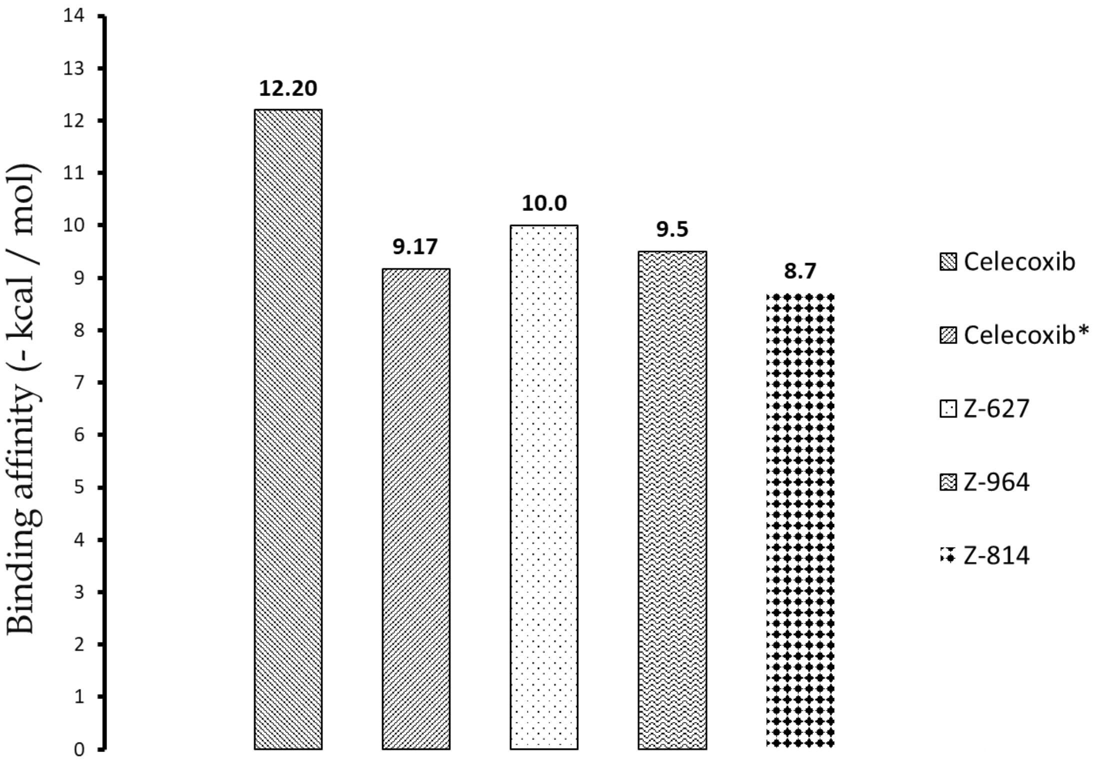

Figure S5. Binding affinity results of compounds, including celecoxib (COX-2 *Mus musculus*). (*Values calculated form experimentally determined constants (K_i). $\Delta G = -RT\ln K_i$, were R (gas constant): $1.987.10^{-3}$ kcal.mol^{-1}.K^{-1}, Temperature: 310 K for celecoxib and K_i: 340.10^{-9} M for celecoxib.

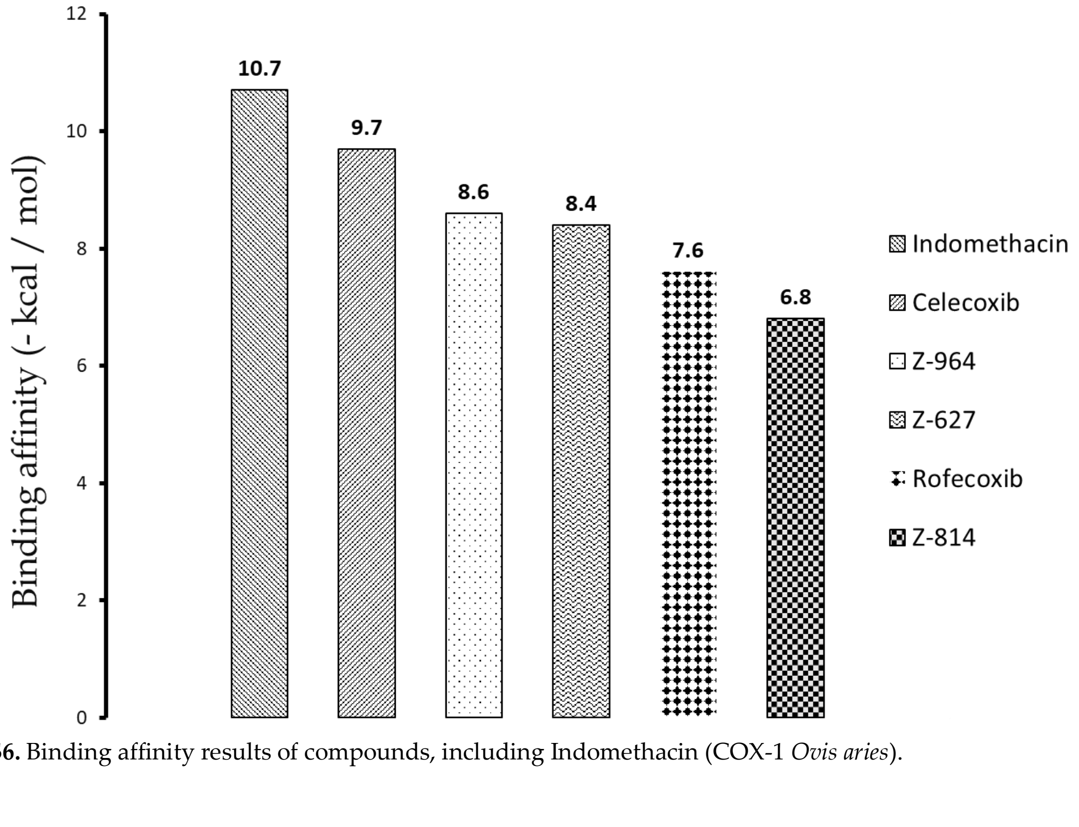

Figure S6. Binding affinity results of compounds, including Indomethacin (COX-1 *Ovis aries*).

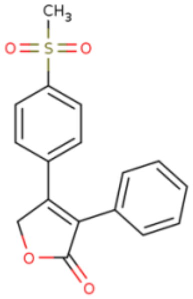
1 (Rofecoxib)
IC_{50} = 12.00 nM

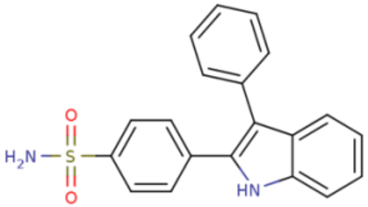
2
IC_{50} = 0.09 nM

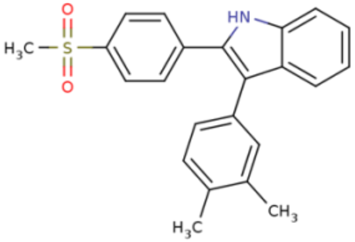
3
IC_{50} = 0.17 nM

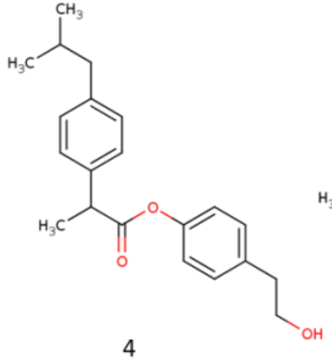
4
IC_{50} = 0.20 nM

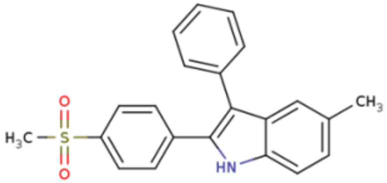
5
IC_{50} = 0.28 nM

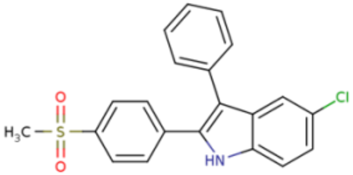
6
IC_{50} = 0.36 nM

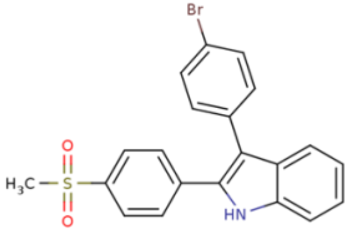
7
IC_{50} = 0.37 nM

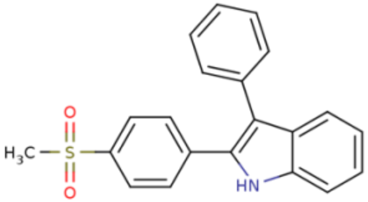
8
IC_{50} = 0.60 nM

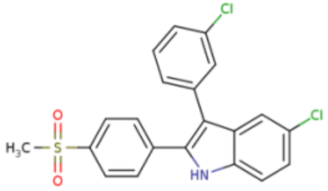
9
IC_{50} = 0.80 nM

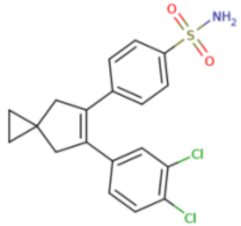
10
IC_{50} = 1.00 nM

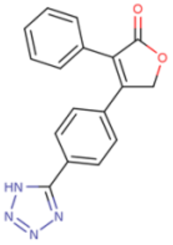
11
IC_{50} = 1.80 nM

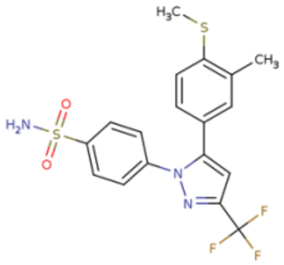
12
IC_{50} = 3.70 nM

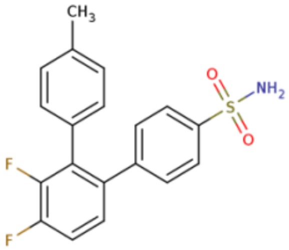
13
IC_{50} = 4.00 nM

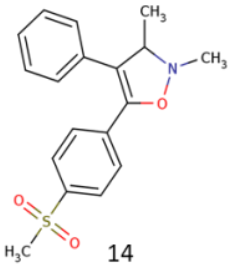
14
IC_{50} = 4.20 nM

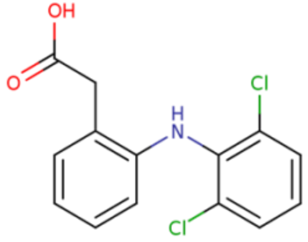
15
IC_{50} = 5.00 nM

Figure S7. Structures used in the molecular modeling.

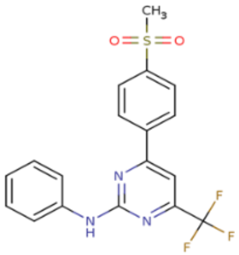

16
IC_{50} = 5.60 nM

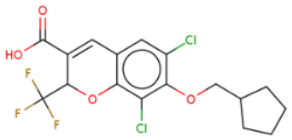

17
IC_{50} = 6.00 nM

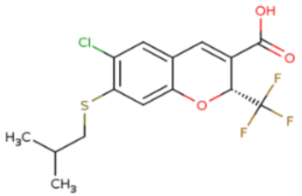

18
IC_{50} = 8.00 nM

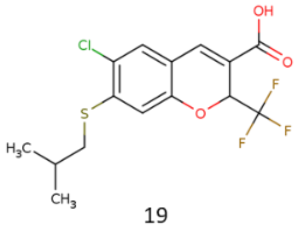

19
IC_{50} = 19.00 nM

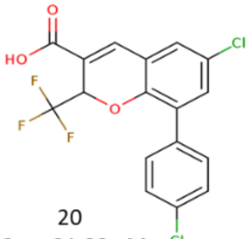

20
IC_{50} = 21.00 nM

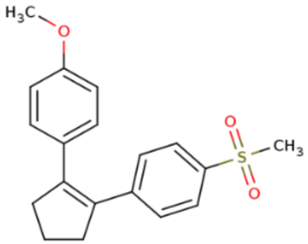

21[a]
IC_{50} = 0.50 nM

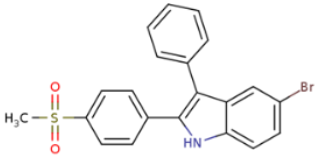

22[a]
IC_{50} = 1.41 nM

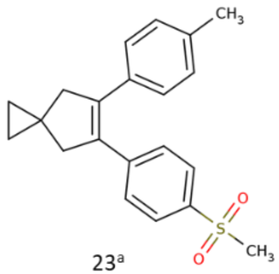

23[a]
IC_{50} = 1.50 nM

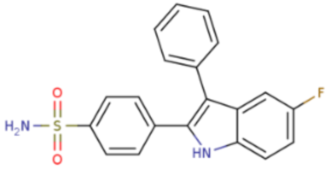

24[a]
IC_{50} = 2.00 nM

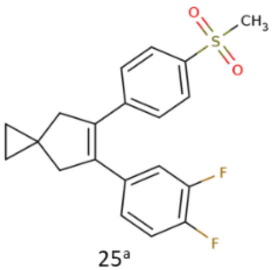

25[a]
IC_{50} = 3.30 nM

Figure S7 *(cont)*. Structures used in the molecular modeling. [a] Internal validation set.

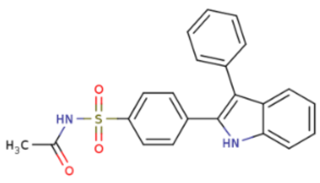

26
IC_{50} = 0.18 nM

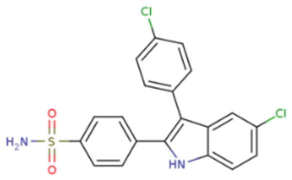

27
IC_{50} = 0.54 nM

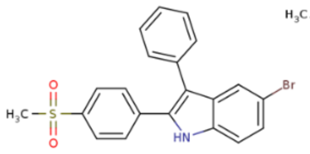

28
IC_{50} = 1.41 nM

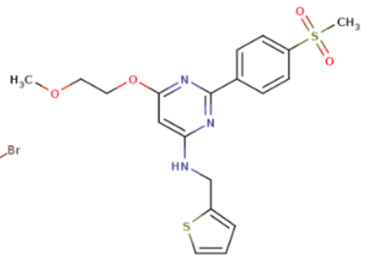

29
IC_{50} = 2.40 nM

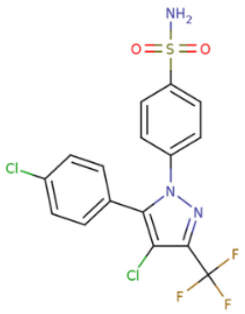

30
IC_{50} = 5.30 nM

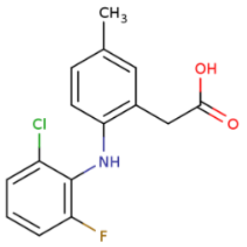

31
IC_{50} = 7.00 nM

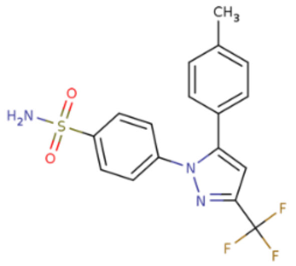

Celecoxib
IC_{50} = 0.52 nM

Figure S8. Structures used in the external validation set.

THEORETICAL SYNTHETIC ROUTES PROPOSED TO COMPOUNDS Z-814, Z-964 and Z-627

Compound A (Z-814):

We propose a theoretical synthetic route **(Figure S9)** for compound **A** based on the formation of phosphonate derivative **III** as intermediate with **I** and **II** as starting materials, sodium hydride (NaH) and *N,N*-dimethylformamide (DMF) as solvent. By an oxidative dephosphorylation reaction[1] under a dioxygen atmosphere, benzyl phosphonate **III** will be used as susbstrate with sodium tert-butoxide (*t*-BuONa) as a base to produce the final symmetrical stilbene **A**.

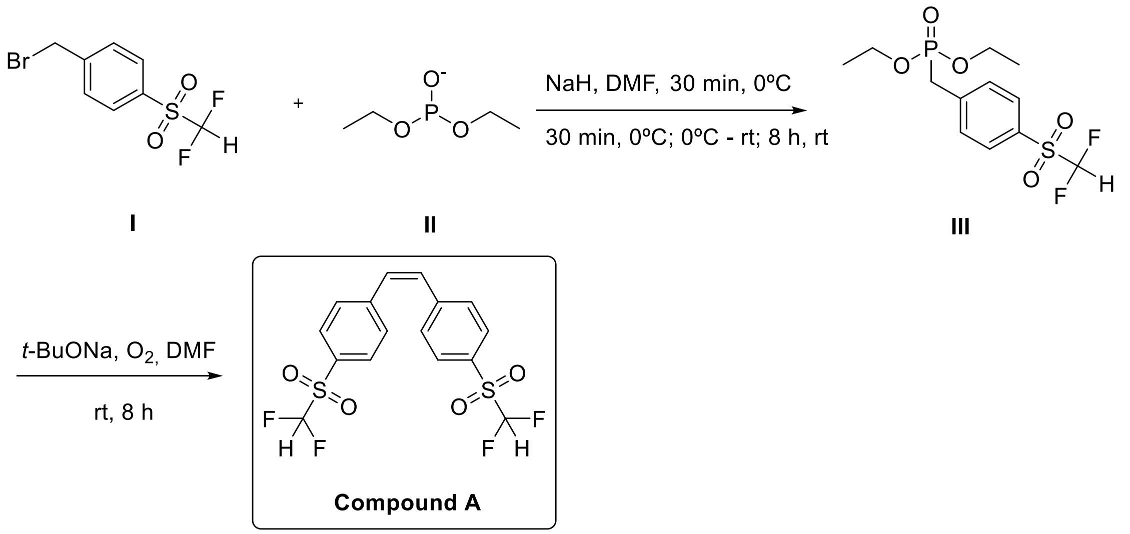

Figure S9. Theoretical synthetic route for the preparation of compound **A** (**Z-814).**

Compound B (Z-964):

Compound **B** can be synthesized through a transamidation reaction using amine **IV** and thiourea **V** as precursors. Solvent-free condition and adding sulfated tungstate to reaction as a catalytic agent[2] will be formed the unsymmetrical thiourea **B (Figure S10)**.

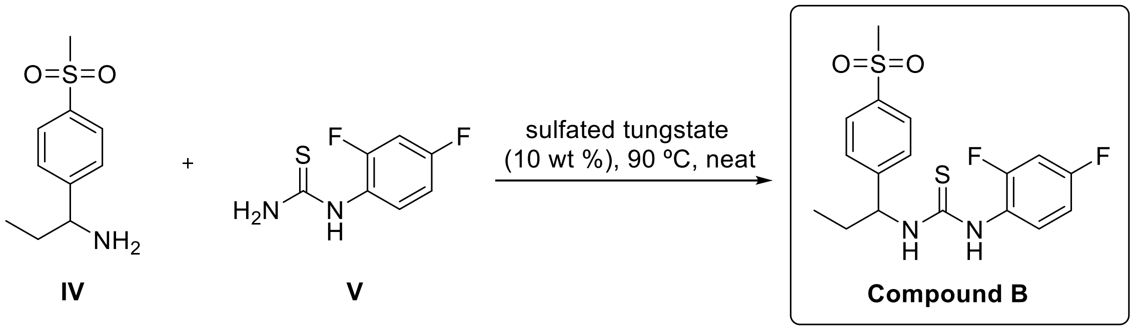

Figure S10. Theoretical synthetic route for the preparation of compound **B (Z-964).**

Compound C (Z-627)

We suggest to use the Ullmann coupling reaction[3] between aryl chloride **VI** and amine derivative **VII** with potassium carbonate (K_2CO_3) as a base, dry diglyme as solvent and using copper as catalyst to give final isoindoline **C (Figure S11)**.

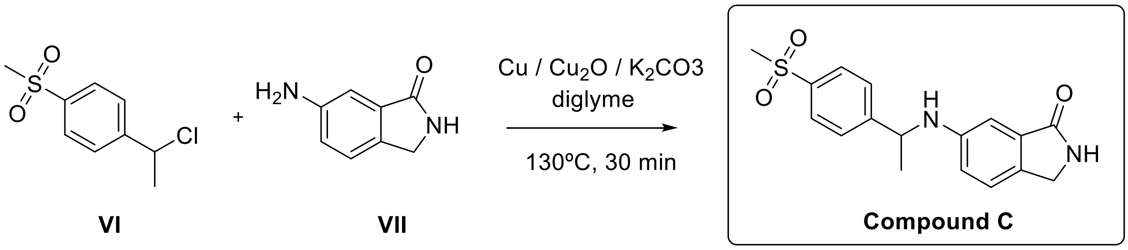

Figure S11. Theoretical synthetic route for the preparation of compound **C (Z-627).**

Note: Starting materials **I, II, IV, V, VI** and **VII** are commercially available.

References

1. Huang, T.; Chen, T.; and Han, L. Oxidative Dephosphorylation of Benzylic Phosphonates with Dioxygen Generating Symmetrical trans-Stilbenes. *J. Org.Chem*. **2018**, *83*, 2959-2965, doi: 10.1021/acs.joc.7b03148.
2. Wagh, G.; Pathare, S.; Akamanchi, K. Sulfated-Tungstate-Catalyzed Synthesis Of Ureas/Thioureas Via Transamidation And Synthesis Of Forchlorofenuron. *ChemistrySelect.* **2018**, *3*, 7049-7053, doi: 10.1002/slct.201800954.
3. Féau, C.; Arnold, L.; Kosinski, A.; Zhu, F.; Connelly, M.; Guy, R. Novel Flufenamic Acid Analogues As Inhibitors Of Androgen Receptor Mediated Transcription. *ACS Chem. Biol.* **2009**, *4*, 834-843, doi: 10.1021/cb900143a
